# Defects mediated weak ferromagnetism in Zn_1−*y*_C_*y*_O (0.00 ≤ *y* ≤ 0.10) nanorods semiconductors for spintronics applications

**DOI:** 10.1038/s41598-023-44102-w

**Published:** 2023-10-10

**Authors:** Saif Ullah Awan, M. Tanveer Akhtar, Danish Hussain, Saqlain A. Shah, Syed Rizwan, Mohsin Rafique, Abdus Samad, M. Arshad

**Affiliations:** 1 Department of Electrical Engineering, NUST College of Electrical and Mechanical Engineering (CEME), National University of Sciences and Technology (NUST), Islamabad, 44000 Pakistan; 2 Department of Physics, COMSATS Institute of Information Technology, Islamabad, 44000 Pakistan; 3 Department of Mechatronics Engineering, NUST College of Electrical and Mechanical Engineering, National University of Sciences and Technology (NUST), Islamabad, 44000 Pakistan; 4 Department of Physics, Forman Christian College (University), Lahore, Pakistan; 5 Physics Characterization and Simulations Lab, School of Natural Sciences (SNS), National University of Sciences and Technology (NUST), Islamabad, 44000 Pakistan; 6 Beijing Academy of Quantum Information Sciences, Beijing, China; 7 Department of Physics, University of Ulsan, Ulsan, 44610 Republic of Korea; 8 Nanosciences 7 Technology Department, National Centre for Physics, Islamabad, Pakistan

**Keywords:** Chemistry, Engineering, Materials science, Nanoscience and technology, Physics

## Abstract

A series of carbon-doped ZnO [Zn_1−y_C_y_O (0.00 ≤ *y* ≤ 0.10)] nanorods were synthesized using a cost-effective low-temperature (85 °C) dip coating technique. X-ray diffractometer scans of the samples revealed the hexagonal structure of the C-doped ZnO samples, except for y = 0.10. XRD analysis confirmed a decrease in the unit cell volume after doping C into the ZnO matrix, likely due to the incorporation of carbon at oxygen sites (CO defects) resulting from ionic size differences. The morphological analysis confirmed the presence of hexagonal-shaped nanorods. X-ray photoelectron spectroscopy identified C–Zn–C bonding, i.e., CO defects, Zn–O–C bond formation, O–C–O bonding, oxygen vacancies, and sp^2^-bonded carbon in the C-doped ZnO structure with different compositions. We analyzed the deconvoluted PL visible broadband emission through fitted Gaussian peaks to estimate various defects for electron transition within the bandgap. Raman spectroscopy confirmed the vibrational modes of each constituent. We observed a stronger room-temperature ferromagnetic nature in the y = 0.02 composition with a magnetization of 0.0018 emu/cc, corresponding to the highest CO defects concentration and the lowest measured bandgap (3.00 eV) compared to other samples. Partial density of states analysis demonstrated that magnetism from carbon is dominant due to its p-orbitals. We anticipate that if carbon substitutes oxygen sites in the ZnO structure, the C-2p orbitals become localized and create two holes at each site, leading to enhanced p–p type interactions and strong spin interactions between carbon atoms and carriers. This phenomenon can stabilize the long-range order of room-temperature ferromagnetism properties for spintronic applications.

## Introduction

Spintronics is an emerging technology that relies on electron spin rather than solely on a charge, as is the case with conventional electronics ^1,2^. This exciting field can potentially develop faster and more efficient computers and memory devices ^3^. Spintronics studies passive control and manipulating spin degrees of freedom in devices ^4^. As one of the hottest frontiers in science and engineering ^5^, spintronics requires a robust platform, similar to silicon technology, which has been the backbone of charge-based electronics. This platform must be constructed using materials and structures that enable the exploration of both charge and spin degrees of freedom for desirable functions ^1^. Consequently, there has been a surge of global research efforts on diluted magnetic semiconductors (DMS) ^1^, particularly ZnO-based systems. According to both the mean-field Zener's model and first-principles calculations, these systems are predicted to exhibit a Curie temperature well above room temperature, provided that the combination of carrier concentration and impurity (magnetic or non-magnetic type) is optimized ^6,7^.

Searching for room temperature ferromagnetic (RTFM) materials is crucial for multifunctional spintronics applications such as spin-valve transistors, spin light-emitting diodes, and nonvolatile storage ^8^. Experimentally, RTFM has been observed in a wide range of oxide materials that do not contain ions with partially filled *d* or *f* bands. This unexpected magnetism is referred to as "* d*^0^ ferromagnetism” ^9,10^ or "defect ferromagnetism", where defects are believed to initiate hybridization at the Fermi level and establish long-range ferromagnetism ^9^. From an application standpoint, ferromagnetic non-magnetically doped ZnO eliminates the possibility of clustering of magnetic impurities, which is a serious drawback for magnetic dopants such as Co, Fe, Ni ^11^ and Mn ^12,13^. Transition metal ions interface formation in ZnO thin films is another possibility of magnetic behavior in these oxide based systems ^14^. Additionally, the optical properties of ZnO that emit UV and visible luminescence of non-magnetic doped ZnO make it a promising multifunctional material with a wide range of potential applications ^15^. To this end, we need to better understand the role of various defects that seem to strongly affect or even control ferromagnetism in this system. It may not be astonishing, as defects have long been recognized to play an essential role in uttering the electrical and optical properties of most wide-bandgap oxide semiconductors. Thus, it is crucial to investigate the roles of non-magnetic dopant defects in the onset of ferromagnetism due to their abundance in wide band gap oxides. Understanding *d*^0^ ferromagnetic materials is a significant challenge. There are two notable features in the magnetic behavior of *d*^0^ ferromagnetic systems: (i) the magnetic moment increases substantially as the temperature is reduced, but (ii) this is caused by an increase in the paramagnetic and not the ferromagnetic component of the magnetic response. There is no clear understanding of this phenomenon. Some researchers have explained it through Zener's model ^8^ and the bound magnetic polaron (BMP) model ^16,17^ while considering both disorder and interactions non-perturbative. However, there is currently no consensus on the origin of ferromagnetism in *d*^0^ systems.

ZnO is a semiconductor ^18^ that has been widely used in multifunctional applications. Ferromagnetism in two-dimensional (2D) nanostructures, such as carbon-doped ZnO thin films, has been reported ^19,20^. However, it is crucial to understand whether one-dimensional (1D) nanostructures, such as nanorods and nanowires of non-magnetically doped ZnO systems, are ferromagnetic. If they are ferromagnetic, which kind of defects should be the origin of the *d*^0^ ferromagnetism? Furthermore, it will be investigated whether that ferromagnetism will be enhanced in 1D nanostructures due to the much larger surface-to-volume ratios compared to 2D nanostructures. Recently, Lin et. al had reported Al doped ZnO nanorods using ultrasonic wave systems ^21^. Since dimensionality affects the band structure and density of states of the systems, further investigations on the magnetic properties of ZnO nanowires are of great importance. We also want to investigate the types of defects and defect densities that promote strong ferromagnetism at or above room temperature. This manuscript will present the structural, electronic, optical and magnetic properties of non-magnetic dopants, e.g., carbon-doped ZnO (known as C-doped ZnO) nanostructures. However, open questions remain related to defect engineering of magnetism in oxides: e.g., what kinds of defects can contribute to magnetic moments? How to establish the long-range magnetic coupling of local moments in an oxide host? These are critical questions that will be addressed in this research article.

The conductivity of ZnO can be varied from insulating to *n*-type semiconductor and even to metallic by substitutional doping with Al or Ga ^22^. However, a major bottleneck for a fully ZnO-based device is the unavailability of *p*-type ZnO. Several groups ^23–25^ have attempted to address this issue unsuccessfully. Mahajan et al. had claimed hole transparent layer of ZnO–WO_3_ nanoparticles for organic solae cells ^26^. The difficulty in *p*-type doping of ZnO arises from native defects, namely oxygen vacancies, unintentionally introduced during synthesis. Native or intrinsic defects are imperfections in the crystal lattice that involve only the constituent elements ^27,28^. These include vacancies, interstitials, and antisites. Native defects can strongly influence the electrical and optical properties of a semiconductor, affecting doping, minority carrier lifetime, and luminescence efficiency and directly impacting diffusion mechanisms, which in turn relate to growth, processing, and device performance. Oxygen vacancies and zinc interstitials have often been considered sources of *n*-type conductivity in ZnO ^27,28^. Compensation effects between charges due to native defects and dopants play a significant role in determining the overall polarity of the medium. The extent and significance of various possible point defects in ZnO are still poorly understood. Most calculations agree that oxygen and zinc vacancies are the lowest energy defects, followed by the Zn interstitial and the Zn_O_ antisite. Oxygen interstitials and O_Zn_ antisites were found to be high in energy. Defects favored under Zn-rich conditions (V_O_, Zn_i_, and Zn_O_) all act as donors, while those favored under O-rich conditions (V_Zn_, O_i_, and O_Zn_) all act as acceptors ^29^. Defect complexes involving more than one such point defect are also possible. The formation energy of point defects controls their concentration in the material. Based on defects, optical bandgap tuning and magnetic behavior of C implanted ZnO thin films ^30^ and N implanted ZnO thin films had been reported by Kumar group systematically ^31^.

One-dimensional non-magnetically C-doped ZnO nanostructures will be fabricated and studied to understand the role of defects in promoting ferromagnetism in them. The primary focus will be identifying how carbon defects (e.g., C_O_) stabilize cation vacancies (V_Zn_) and how these may potentially lead to ferromagnetism. This new class of ferromagnetism, defect-based ferromagnetism, is currently not well understood. The effects of different defect content on various properties will be studied. The impact of size mismatch disorder resulting from doping carbon on the oxygen sites at different concentrations is of particular significance since the interactions between different types of defects tend to affect the disorder. Therefore, a systematic study of such effects on the structural, electronic, magnetic, and optical properties is necessary. The mechanisms of long-range ferromagnetic order will be investigated with the help of computational studies of the samples. This manuscript presents a systematic study of the growth of one-dimensional ZnO nanostructures. The main focus of this study is the development of ferromagnetism in one-dimensional non-magnetically doped ZnO nanostructures and the role defects play in this regard. While theoretical studies of such 1-D carbon-doped ZnO nanostructure systems do exist ^32,33^, the specific question of which type of defect—substitutional or interstitial—plays a significant role in stabilizing ferromagnetism and how that is to be explained consistently has not been experimentally performed over a significant range of carbon concentrations. Consequently, questions arise regarding how carbon helps stabilize the zinc vacancy. In the substituent role, carbon is understood to be a hole dopant. So, how does this affect the stabilization of ferromagnetism in the respective situation? While both carbon and zinc vacancies can be considered hole dopants, oxygen vacancies, another major defect in these systems, act as electron donors. How do these competing tendencies affect the behavior?

## Experimental section

### Hydrothermal process

Oxides-based semiconducting nanorods and nanowires can be synthesized by well know hydrothermal techniques ^34^. This process comprises two steps: (a) seed layer preparation via spin coating and (b) nanostructure growth via dip coating process.

#### Synthesis of seed layers on Si substrate through spin coater

**Figure 1:**
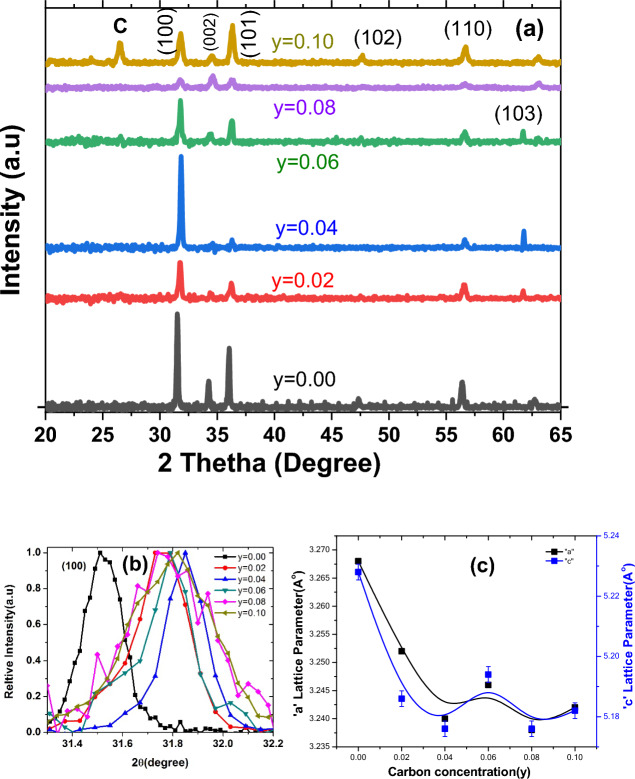
(**a**) XRD pattern of Zn_1−*y*_C_*y*_O (0.00 ≤ *y* ≤ 0.10) nanorods, (**b**) 2θ shifting of (100) plane at relative intensity for entire series of samples, and (**c**) variation in the lattice parameters with increasing the C content.

First, silicon (Si) substrates (001) were cleaned with acetone, 1-propanol, and distilled water for 10 min using a ultrasonic bath. After washing, the Si substrates were dried in the oven. We have used zinc acetate (C_4_H_6_O_4_Zn.2H_2_O) and 1-propanol (C_3_H_8_O) precursors for the preparation of 5 mM solution for the synthesis of non-uniform thin layers (known as seeds). This solution (Zinc acetate + 1-propanol) is called a “seeded solution.” A non-uniform thin layer of seeded solution was deposited on the Si substrate using a spin coater. After spin coating, the seed layers solution deposited Si substrates were annealed at 350 °C for 20 min. This non-uniform thin layer is called “seed layer,” and substrates are now called “seeded Si” substrates. We have synthesized seed layers on bare Si substrate to understand how many layers will be helpful for the growth of ZnO nanostructures. Figure 1S(a) shows the SEM image of the Si bare substrate, which has a clean surface. Figure 1S(b–d) presents SEM images of 1-layer (one-time coated), 2-layer (two-time coated), and 3-layers (three-time coated), indicating the discontinuous distribution of seeds are present on Si substrate except 1-layer Si coated. So, in our case, we found that one time coated (known as 1-layer) of seeds is suitable for the growth of ZnO nanorods structures. The growth structure of these coated layers depends on the temperature, i.e., 85 °C and the thickness of seed layers. We used a single layer of seeds on the Si substrate for all compositions of samples. Seed layers play a role in the nucleation sites.

These seed layers promote nucleation for the growth of nanostructures (nanowires/nanorods) due to lowering the thermodynamic barrier. Seed layers assume a vital part in diverse conditions. Seeds can enhance the arrangement and length of nanorods/nanowires at distinctive temperatures and conditions. Growth-rate is very little enhanced at settled seed annealing temperature. The growth of nanorods at the silicon-seeded substrate indicated better crystallinity. Seed of the least layers has more emotional results than expansive layers to make precise nanostructures ^35^.

#### Synthesis of Zn_1−y_C_y_O (0.00 ≤ ***y*** ≤ 0.10) nanorods on “Seeded Si” substrate via dip coating

A series of C-doped ZnO [Zn_1−y_C_y_O (0.00 ≤ y ≤ 0.10)] samples were grown on seed Si substrate. We prepared an equal molar 20 mM solution of zinc acetate and hexamethylenetetramine (HMTA) in deionized (DI) water. The precursors were completely dissolved in DI water via a sonicator for 30 min. This solution was used for the synthesis of pure ZnO samples. For doping the carbon into the matrix of the ZnO structure, we used graphite (a carbon “C”) powder and prepared a 20 mM solution in DI water. In this solution, the concentration of C-doping was established as y = 0.02, 0.04, 0.06, 0.08, and 0.10 compositions. Carbon was not completely dissolved in DI water after sonicating for 1 h, but carbon may act as a suspended solution with DI water. We have mixed the pure and carbon solutions to synthesize C-doped ZnO samples. We have placed the seeded Si substrates in the mixture solution known as the dip coating process. We have placed this solution on a hot magnetic plate. We provide continual heat at 85 °C for 3 h. Silicon oil was used in a bowl to provide homogeneous heat during growth.

The use of Hexamethylene–tetramine (HMTA) and zinc nitrate hexahydrate as chemical mediators for developing ZnO nanostructures is a common technique described by many researchers ^36^. This method uses zinc nitrate salt to maintain Zn^2+^ ions, while O^2−^ molecules in the solution maintain ions. The exact role of HMTA in the growth of ZnO nanowires/nanorods is not yet clear. However, it is believed to slowly hydrolyze in weakly basic conditions and provides OH– ions. However, the technique presents a challenge due to the rapid hydrolysis of HMTA, which produces many OH– ions quickly. This results in the precipitation of Zn^2+^ ions in the high pH environment, leading to a limited contribution to the growth of ZnO nanowires and the rapid consumption of the supplement, preventing further growth of ZnO nanowires. These observations can be explained by five different reactions ^37^:

Decomposition-reaction:

$$\left( {{\text{CH}}_{{2}} } \right){\text{6N}}_{{4}} + {\text{6H}}_{{2}} {\text{O}} \leftrightarrow {\text{4NH}}_{{3}} + {\text{6HCHO}}$$

Hydroxyl supply-reaction:

$$\begin{aligned} {\text{NH}}_{{3}} + {\text{H}}_{{2}} {\text{O}} & \leftrightarrow {\text{NH}}_{{3}} \cdot {\text{H}}_{{2}} {\text{O}} \\ {\text{NH}}_{{3}} \cdot {\text{H}}_{{2}} {\text{O}} & \leftrightarrow {\text{NH}}^{{{4} + }} + {\text{OH}}^{ - } \\ \end{aligned}$$

Supersaturation-reaction:

$${\text{Zn}}^{2 + } + 2{\text{OH}}^{ - } \leftrightarrow {\text{Zn}}\left( {{\text{OH}}} \right)_{2}$$

ZnO Nanorods growth-reaction:

$${\text{Zn}}\left( {{\text{OH}}} \right)_{{2}} \to {\text{ZnO}} + {\text{H}}_{{2}} {\text{O}}$$

The growth mechanism of the ZnO nanorods can be organized through these five reactions. Most of the reactions in equilibrium can be controlled by reaction parameters, such as precursor concentration, growth temperature, and growth time ^37^.

### Characterizations performed

The X-ray diffraction (XRD) technique was used to examine the phase of the prepared compounds. An X-ray diffractometer (PANalytical X-pert pro) with Cu K-Alpha radiation (λ = 1.5406 Å) was employed. The morphology of fabricated nanorods was examined by scanning electron microscope (Hitachi S-4800 microscope operated at 20 kV). X-ray photoelectron spectroscopy (XPS) data was obtained using a Scienta-Omicron system with a micro-focused monochromatic Al K-Alpha X-ray source. The x-ray source was operated at 15 keV with 700-micron spot size, constant analyzer energy (CAE) 100 eV for survey and 20 eV for detailed scans. The Charge neutralization was applied using a combined low energy/ion flood source to avoid the charging effects. The data acquisition was performed with Matrix software, and data analysis was performed with Igor Pro and XPS fit procedures. The curve fitting of detailed spectra was done using a Gaussian–Lorentzian line shape after performing the shrilly background corrections. The C1s binding energy fixed at 284.5 eV was used for referencing the data. Raman spectra were recorded using a Laser Micro Raman spectrometer (LabRamHR) with an excitation source having a wavelength of 514.5 nm. Room Temperature PL spectra were measured with RF-5301 PC Fluorescence Spectro-fluoro-photometer. Magnetic measurements were performed at 300 K using a Vibrating Sample Magnetometer (VSM) Model-740 of Lakeshore (USA).

## Results and discussion

### Structural properties

**Figure 2:**
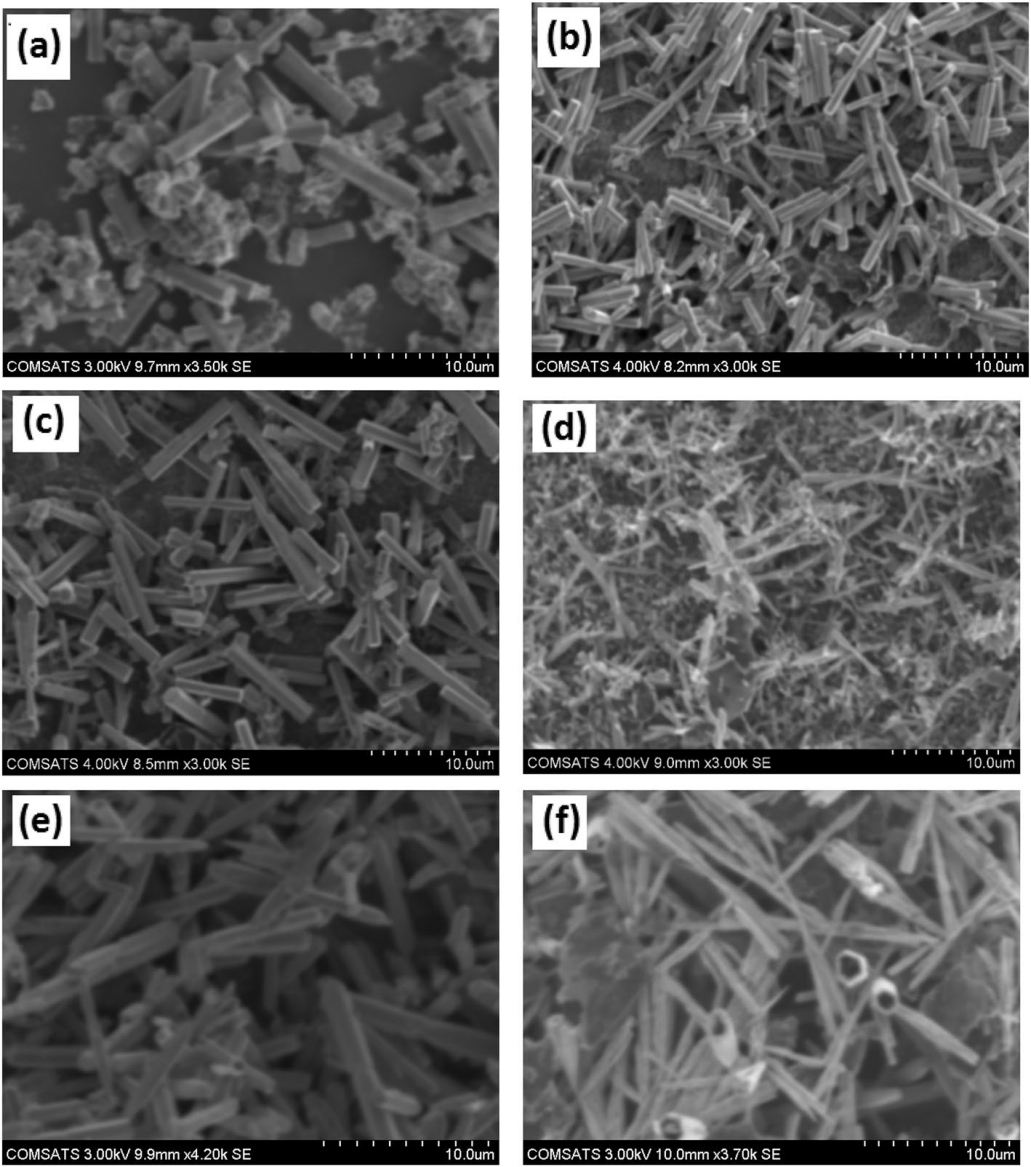
High resolution (i.e. 10 μm scale) SEM images of (**a**) pure, (**b**) 2%, ZnO, (**c**) 4% ZnO, (**d**) 6%, (**e**) 8%, and (**f**) 10% C-doped ZnO nanorods.

X-ray diffraction (XRD) measurements were performed for the entire series of Zn_1−y_C_y_O (0.00 ≤ y ≤ 0.10) samples, as presented in Fig. 1a. The diffracted XRD peaks of all samples included the majority of planes (100), (002), (101), (102), (110), and (103), corresponding to the hexagonal ZnO crystal structure ^38–40^. JCPDS (Joint Committee on Powder Diffraction Standards) file No. (JCPDS: No. 01-075-0576). Similar hexagonal crystal structure of ZnO doped with Ag had been reported recently ^41^. We successfully achieved a single phase in doped samples and did not observe any secondary phase up to the limit of y = 0.08 concentration. However, upon further carbon doping, we found an impurity peak corresponding to graphite or carbon for the y = 0.10 sample. We noticed that the intensity variations among relative and corresponding peaks in all samples depend upon the preferred growth orientation, as shown in Fig. 2S(a). However, we observed that the overall intensity of the undoped ZnO sample is higher compared to doped samples, indicating that the crystal quality of the undoped sample is higher and that the dopant C decreases the crystal quality of the ZnO structure. We observed a shift in the 2θ values toward higher angles in carbon-doped samples compared to the undoped ZnO sample. This shift in prominent peaks (100), (002), and (101) is demonstrated in Fig. 2S(a) at ordinary intensity. We plotted the relative intensity of prominent peaks, while the shift in the most intense prominent peak (100) is shown in Fig. 1b. The significant shift in the 2θ values indicates that the carbon dopant has been incorporated into the matrix of ZnO nanostructures. The measured and calculated values of diffracted peak positions for prominent planes, *d*-values, lattice parameters, unit cell volume, and crystallite size of nanostructured samples from XRD data are presented in Table 1S. Depending on the concentration and distribution of carbon atoms, the crystallite size of ZnO may increase or decrease compared to the pristine material. Regarding lattice parameter changes, the introduction of carbon atoms can induce lattice strain in the ZnO host matrix. The specific changes in crystallite size and lattice parameter will depend on various factors, including the concentration and distribution of carbon atoms (may occupy interstitial sites or substitute), the crystal structure of ZnO, and the synthesis conditions employed. Additionally, carbon atoms can induce lattice strain and alter the lattice parameter of ZnO. The variation in lattice parameters with increasing C content is plotted in Fig. 1c. We noticed that the lattice parameter decreased with increasing C content in doped samples. Similarly, a decreasing trend of unit cell volume versus C concentration is plotted in Fig. 2S(b).

Computational studies ^19,20,36,42^ have predicted that hole carriers can be created when C substitutes O sites. By comparing the ionic sizes of Zn^2+^ (0.074 nm), O^2−^ (0.121 nm), and C^4+^ (0.029 nm), as the C ions occupy the O sites, the unit cell volume will contract (compress). Consequently, the lattice parameters of the hexagonal ZnO system will decrease, and ultimately, 2θ values of diffracted peaks will shift toward higher angle values. This observation indicates a decrease in lattice constant with carbon doping, which supports the prediction that carbon ions are incorporated into the ZnO structure, and that C can replace O lattice sites, as previously reported ^19,42–44^. In doped samples, from the XRD data, we may infer that C substitutes O sites, as previously reported by other computational studies ^19,42^, for the mediation of hole carriers in ZnO systems. Generally, we achieved a single-phase hexagonal (wurtzite) structure, except for y = 0.10. We observed that lattice parameters of doped samples decreased with increasing C concentration. Based on the ionic sizes of the dopant and host material, the unit cell volume will decrease as computationally expected when C substitutes O sites.

### Microstructure morphology

**Figure 3:**
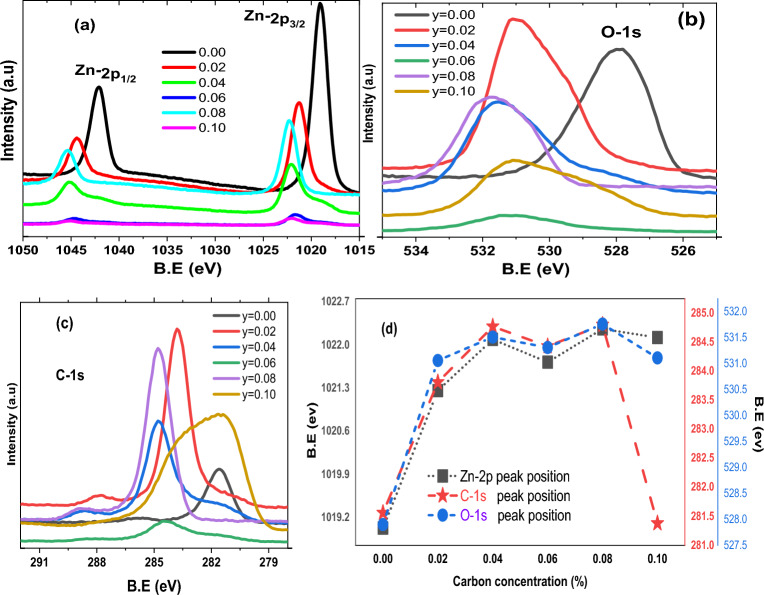
High resolution XPS core level spectra of a series Zn_1−*y*_C_*y*_O (0.00 ≤ *y* ≤ 0.10) nanorods (**a**) Zn-2p (**b**) O-1 s (**c**) C-1 s (d) variation of binding energies of prominent Zn-2p, O-1 s, C-1 s versus Carbon concentration in ZnO nanorods samples.

Scanning electron microscope (SEM) measurements were performed to investigate the morphology and dimensions. The SEM micrographs of the entire series of Zn_1−y_C_y_O (0.00 ≤ y ≤ 0.10) nanorods at 50 µm scale ranges are shown in Fig. 3S(a–f). These images demonstrate that all samples exhibit well-controlled one-dimensional (1D) nanorod-type morphology. More precisely, Fig. 2a–f clearly shows the 1D morphology of all samples at a higher range, with a 10 µm scale range captured by SEM and nanorods' dimensions measured by the software "ImageJ." In the pure ZnO sample (Fig. 2a), the dimensions of hollow microrods are as follows: the average length is 23.056 µm, the outer diameter is 3.92 µm, and the inner diameter is 1.14 µm. The aspect ratio of these hollow nanorods is approximately 3.43. Similar nanostructured morphologies (e.g., tubes/rods/wires) of ZnO systems have been reported earlier ^45^. High-resolution SEM micrographs of the 2% C-doped ZnO sample are presented in Fig. 2b, showing that the rods are highly dense and not hollow. We found that the rods have a randomly oriented distribution in lateral positions. The hexagonal crystallite structure is more apparent in the highest-resolution image. The average length of rods is 9.915 µm, and the diameter is approximately 1.5 µm. The aspect ratios of these structures are approximately 6.61. Figure 2c shows fine crystalline structures and random orientation with the hexagonal wurtzite-like structure for the x = 0.04 sample. The highest-resolution image displays the average length of the rod at 8.48 µm and the average diameter at approximately 1.4 µm. The aspect ratios of these structures are approximately 6.06. Figure 2d represents the maximum length of any nanorod as 7.02 µm and the diameter as approximately 800 nm. The aspect ratios of these structures are approximately 8.7 µm. Figure 2e demonstrates that the x = 0.08 sample had a maximum nanorod length of 15.67 µm, a diameter of approximately 900 nm, and aspect ratios of these structures as approximately 17.41 µm. Figure 2f shows that 10% carbon-doped ZnO has mixed structures (nanorods, nanoneedles, nanotubes, and nanowires). The average length of nanostructures is 14 µm, and the outer diameter of hollow nanorods is approximately 2.2 µm. The inner diameter is approximately 900 nm, with an aspect ratio of these structures at approximately 6.56. Overall, from the morphological results of the C-doped ZnO series, we concluded that in pure ZnO samples and 10% C-doped ZnO samples (in this sample, C was not appropriately doped into the matrix of ZnO), we achieved hollow rod-shaped structures. In contrast, all other C-doped samples displayed dense-filled rod/wire-shaped structures in the SEM images. Overall, we achieved high-density growth of nanorods in C-doped ZnO samples on seeded Si substrates. The length and diameters of these nanorods varied; however, we obtained hexagonal morphology.

### X-ray photoelectron spectroscopy (XPS)

**Figure 4:**
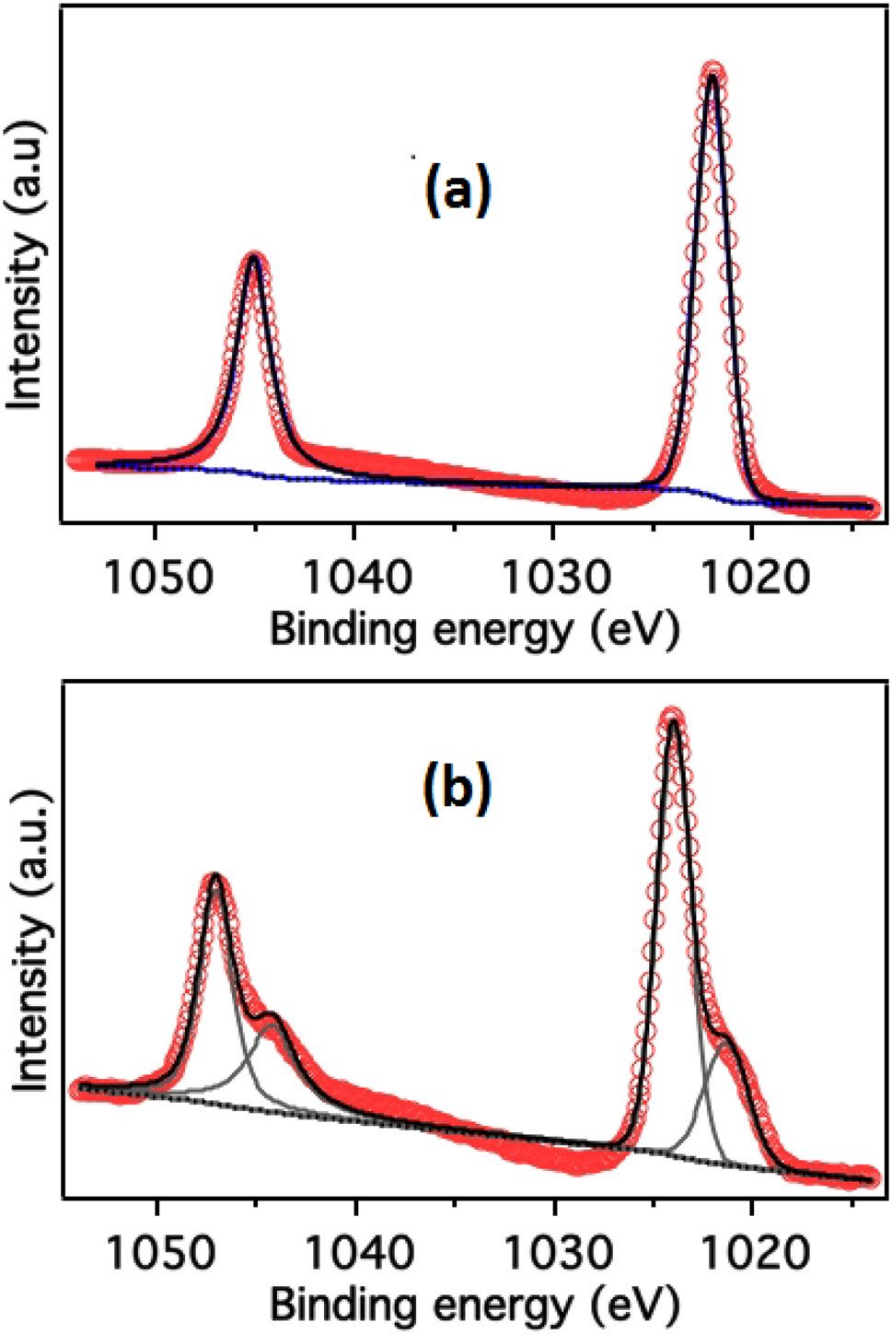
High resolution Zn-2p XPS core level spectra of (**a**) y = 0.00 and (**b**) y = 0.10 nanorods samples.

The surface analysis of for entire series of Zn_1_-_y_C_y_O (0.00 ≤ y ≤ 0.10) samples is performed by XPS. A Low-resolution, wide-ranging XPS survey scan for entire series of nanorod samples is demonstrated in Fig. 4S. The survey spectra obtained from prepared samples show the presence of zinc (Zn), oxygen (O), and carbon (C) only due to major constituents, and the presence of Si-2p confirmed samples were deposited on Si substrate. In our data, complete detected peaks can be attributed to Zn, O, and C with their corresponding Auger peaks as categorized in Fig. 4S. We did not find any core transition metal elements (e.g., cobalt or iron) within the detection limit of our XPS technique. Our EDX (energy dispersive X-Ray) results (data not shown) were obtained during SEM measurements of nanorod samples. They revealed similar results as XPS data replicates. These evident results confirm that our nanorod samples contain predominantly Zn, O, and C species. We obtained high-resolution core-level spectra of Zn-2p, O-1 s, and C-1 s, as revealed in Fig. 3a–c, respectively, for the entire series of samples. Interestingly, we found that the binding energies of all these constituents are shifted with respect to each other. We noticed conspicuous variation in binding energies of prominent peaks of Zn-2p, O-1 s, and C-1 s versus carbon concentration in ZnO nanorods samples, as presented in Fig. 3d that confirmed the chemical changes that occurred after doping C in ZnO structure. Remarkably, we found the binding energies values of each constituent shifted towards higher values as increasing the C concentration is consistent except y = 0.10 sample. We may infer that C doping affects the common chemical bonding of Zn and O after incorporating it in a hexagonal crystal structure.

Our core spectra Zn-2p of y = 0.00, y = 0.02, and y = 0.08 is symmetric while y = 0.04, y = 0.06, and y = 0.10 have asymmetric nature as clearly visible in Fig. 3a. The deconvoluted high-resolution Zn2p core-level photoemission spectra received from a few selected samples, e.g., pristine ZnO (y = 0.00) and y = 0.10 samples, are shown in Fig. 4a,b. For undoped ZnO, the Zn2p_3/2_ and 2p_1/2_ core level places are leveled at 1022.0 eV and 1045.1 eV, which are attributed to the occurrence of zinc in Zn^2+^ the oxidation respectively ^46^ and attained tetrahedral coordinated on the wurtzite structure surrounded by O^2−^ atoms ^47^. For the undoped ZnO samples, there are single sharp peaks for Zn2p (3/2, 1/2) core levels; conversely, for Zn_1−y_C_y_O (y = 0.10) peaks are broadened along with additional features which suggest the change of chemical composition at the surface of the material. For the Zn_1−y_C_y_O (y = 0.10) sample, the Zn2p_3/2_ core level is deconvoluted into two peaks. The peak at a binding energy of 1023.9 eV is assigned to Zn^2+^ in the ZnO wurtzite structure, and the second peak at 1021.5 eV characterizes the zinc hydroxide species ^48^. On the electronegativity difference between O (χ_O_ = 3.44) and C (χ_C_ = 2.55), our former fitted peak shifted to higher energy centered at ~ 1023.9 eV might be associated with Zn^2+^ and the fitted peak at lower energy side correspond to Zn–oxy–carbide (Zn–O–C) bonds ^47,49^, due to the considerable difference in binding energy through the Zn–O–Zn peak (2.40 eV in our case).

**Figure 5:**
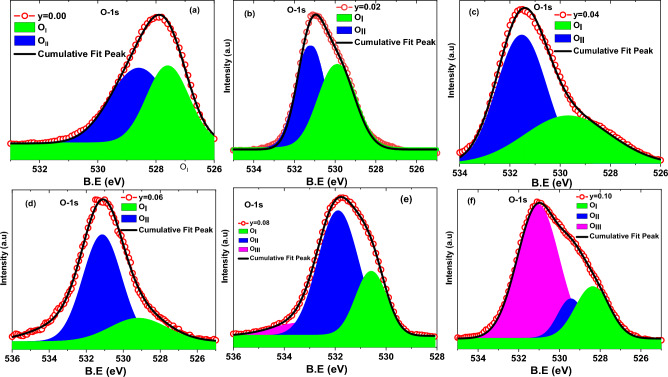
High resolution O-1 s XPS core level spectra of a series Zn_1−*y*_C_*y*_O (0.00 ≤ *y* ≤ 0.10) nanorods samples.

Figure 3b presents asymmetric nature of the high resolution core-level O-1 s spectra of entire series of C-doped ZnO nanorods samples. The deconvoluted O-1 s spectrum of Zn_1−y_C_y_O (0.00 ≤ y ≤ 0.10) illustrated in Fig. 5a–f. The O-1 s asymmetric spectrum of each nanorod sample is well fitted and deconvoluted into two peaks for y = 0.00, y = 0.02, y = 0.04, y = 0.06, while three peaks for y = 0.08, y = 0.10 concentrations. The first fitted peak at the lower binding energy side (in our case O_I_), usually centered at 530(± 0.5) eV corresponds to O^2−^ ions bounded by Zn in the ZnO lattice indicating an oxidized atmosphere, while the second fitted peak (in our case O_II_) placed at 531(± 0.5) eV is typically associated with oxygen vacancies (V_O_) within ZnO matrix ^50^ or here in our C-doped ZnO case may be due to C bonded with Zn through O species. This O_II_ component can be thought of as principally linked to Zn–O–C bonds, as reported previously ^47^. We may argue here that in pure ZnO system, OII component usually represents oxygen vacancies, but in C-doped ZnO systems, those oxygen vacancies may be filled with C-doped atoms due to C doping. The third fitted peak (in our case O_III_) at 532(± 0.5) eV characterizes the zinc bonding with hydroxyl species ^51^.

**Figure 6:**
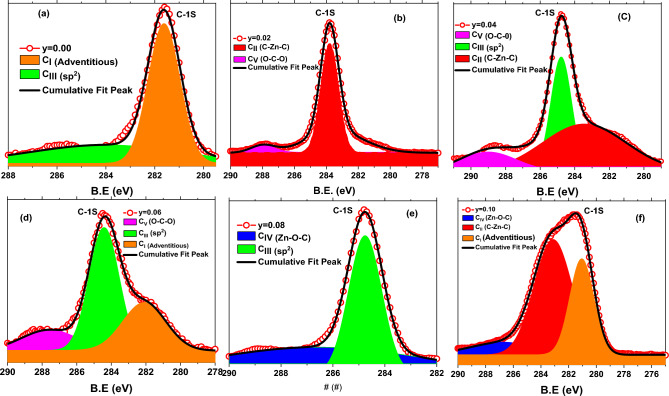
Deconvoluted C-1 s XPS core level spectra of a series Zn_1−*y*_C_*y*_O (0.00 ≤ *y* ≤ 0.10) nanorods samples.

Asymmetric type high-resolution core-level C-1 s spectra of entire series of C-doped ZnO nanorods samples had revealed in Fig. 3c. Furthermore, in Fig. 6a–f, the deconvoluted C-1 s spectrum of Zn_1_-_y_C_y_O (0.00 ≤ y ≤ 0.10) samples was established to understand and estimate the defects created in the C-doped ZnO system. Like wisely, in Fig. 6a, the C-1 s core level from the undoped ZnO nanorod sample is resolved into two components located around ~ 281.62 eV (C_I_), and ~ 284.78 eV (C_III_) are associated with adventitious carbon and sp^2^ hybridization respectively, however in pure ZnO the deconvoluted peak (C_III_) also associated with C–OH/C–O–C and C=O. Furthermore, the C-1 s spectrum received from C-doped ZnO samples (Fig. 6b–f) is deconvoluted into two or three fitted sub-peaks, which are positioned at 281.62 (± 0.5) eV (C_I_), 283.79 (± 0.5) eV (C_II_), 284.78 (± 0.5) eV (C_III_), 286.60 (± 0.5) eV (C_Iv_), 287.52 (± 0.5) eV (C_v_). The deconvoluted peak at centered 281.62 (± 0.5) eV (C_I_ of orange color shaded area) is usually arise and we may refer as adventitious contamination. The fitted peak situated at 283.79 (± 0.5) eV (C_II_ of red color shaded area) corresponds to the carbon bound to Zn and assigned with C–Zn–C bonding, i.e., ***C***_***O***_ in the ZnO structure. The C-1 s sub-peak at position 284.78 (± 0.5) eV (C_III_ of green color shaded area) is consigned to free graphitic carbon (i.e., sp^2^-bonded carbon). The deconvoluted peak at location 286.60 (± 0.5) eV (C_IV_ blue color shaded area) is assigned with zinc oxy-carbide complex and linked with Zn–O-C bond formation. Another fitted peak pinpointed at 287.52 (± 0.5) eV (C_V_ of magenta color shaded area) is attributed to carbon–oxygen bonds and allocated with O–C–O bonding or with C–OH, C>=O, and COOH, carbonyl group respectively. These allocations agree with the literature as well ^47,49,52–56^.

### Raman spectroscopy

Raman scattering was used to analyze the vibrational characteristics of the ZnO nanorods. Hexagonal (wurtzite) ZnO nanostructures are related to the space group (P63mc). The Brillouin zone describes vibrational modes based on group theory; Γopt = A1 + 2B1 + E1 + 2E2 ^57–60^. Here, E1, E2, and A1 are the Raman active modes. E1 and A1 are also infrared-active and polar. B1 is prohibited. Thus, A1 and E1 are divided into transverse optical (TO) and longitudinal optical (LO) ^57,60,61^. Typically, second-order spectra are further divided into three regions: (I) low-frequency region (160–540 cm^−1^ approximately) formed by acoustic overtones, (II) high-frequency region (820–1120 cm^−1^ approximately) dominated by optical overtones and combinations, and (III) intermediate-frequency region (540–820 cm^−1^) dominated by optical and acoustic phonon combinations ^62^. The modes at 576, 1151, 1735, and 2326 are related to the scattering from the first and higher-order LO phonons ^63^. The peaks A1(LO) and E1(LO) positioned at approximately 584 cm^−1^ describe the formation of defects such as oxygen vacancies, interstitial Zn, and the lack of free carriers ^64^.

**Figure 7:**
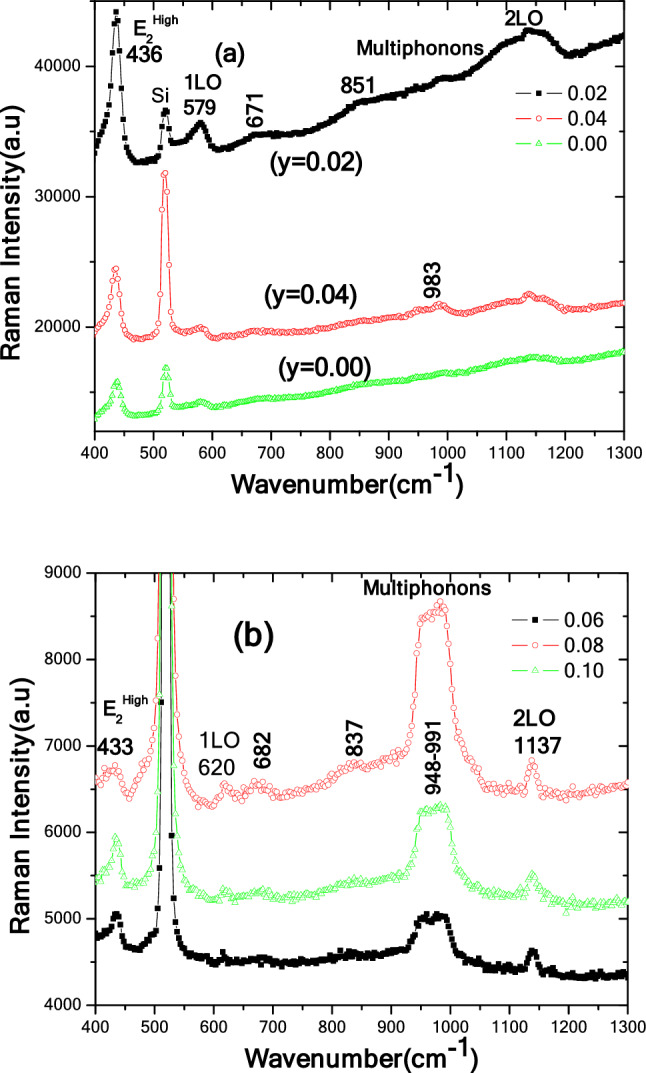
Room temperature Raman spectra of Zn_1−*y*_C_*y*_O (0.00 ≤ *y* ≤ 0.10) nanorods. (**a**) Active vibrational modes *y* = 0.00%, 0.02%, and 0.04%. (**b**) Active vibrational modes *y* = 0.06%, 0.08%, and 0.10% samples.

Room temperature Raman spectra of Zn_1_-_y_C_y_O (0.00 ≤ y ≤ 0.10) samples in the spectral range of 400–1300 cm^−1^ are presented in Fig. 7a,b. We conducted Raman measurements in a backscattering configuration. Table 2Sdisplays the relevant positions of all possible modes in the nanorod samples. We further divided our data into two groups, as shown in Fig. 7a,b. We observed different modes at various frequencies, as reported in the literature ^62,65^, for this series of C-doped ZnO samples on the seeded Si substrate. In Fig. 7a,b, we observed a highly intense Raman mode at 520 cm^−1^, corresponding to the vibrations of Si lattice atoms. The second prominent mode appeared in samples at 436 cm^−1^, corresponding to E2High vibrations. The Raman E2High peak is an active, sharp, non-polar optical phonon mode related to oxygen vibrations ^62,65^. We did not observe the E2Low mode in our data due to the limitations of our instrument. This E2Low mode, usually obtained at 100 cm^−1^, corresponds to Zn vibrations.

We observed the A1(LO) mode at 579 cm^−1^ in y = 0.0%, y = 0.02%, and y = 0.04% samples. In contrast, we detected the TA + TO mode at position 620 cm^−1^ in y = 0.06%, y = 0.08%, and y = 0.10% doped samples. This significant change observed in the LO mode indicates a notable alteration in the lattice vibrations and phonon behavior of our system. The LO mode corresponds to the vibration of the crystal lattice along the direction of the incident light. This mode is sensitive to changes in the crystal structure, strain, doping, defects and compositions that can affect the phonon behavior of the material can contribute to these shifts in the Raman peaks, indicating modifications in the local environment and lattice properties of the material. However, we noticed the TA + LO mode at 671 cm^−1^ in y = 0.0%, y = 0.02%, and y = 0.04% samples, while the TA + LO mode at 851 cm^−1^ was observed only in the y = 0.0% sample. Similarly, these two TA + LO modes were present in y = 0.06%, y = 0.08%, and y = 0.10% samples at 682 cm^−1^ and 837 cm^−1^. As shown in Fig. 7a,b, we identified a long-range band in the 943–991 cm^−1^ spectral range. This mode may be related to carbon-induced multi-phonons since multi-phonons are absent in the undoped ZnO sample. Such multi-phonons are developed due to the disorder in ZnO structures caused by C species doping. We fixed the central position of this mode at 983 cm^−1^, corresponding to 2TO. Additionally, we observed the 2LO mode at 1140 cm^−1^ in y = 0.00%, y = 0.02%, and y = 0.04% samples, and at 1137 cm^−1^ in y = 0.06%, y = 0.08%, and y = 0.10% samples. Furthermore, by comparing the Raman intensity of one of the prominent modes (e.g., E2High), we found that the y = 0.02% and y = 0.04% doped samples had sharp peaks, while y = 0.06%, y = 0.08%, and y = 0.10% doped samples exhibited less intense peaks than the undoped ZnO nanorod sample. This observation suggests good incorporation of C atoms in ZnO structures. Overall, room temperature Raman backscattering spectra of C-doped ZnO samples showed different modes at various frequencies. While E_2_^High^, A1(LO), TA + TO, TA + LO, and 2LO modes were present in all samples. The 2TO mode at 983 cm^−1^ was present in C-doped samples, corresponding to multi-phonons, potentially due to C species. This mode was absent in the pure ZnO sample.

### Photoluminescence spectroscopy

**Figure 8:**
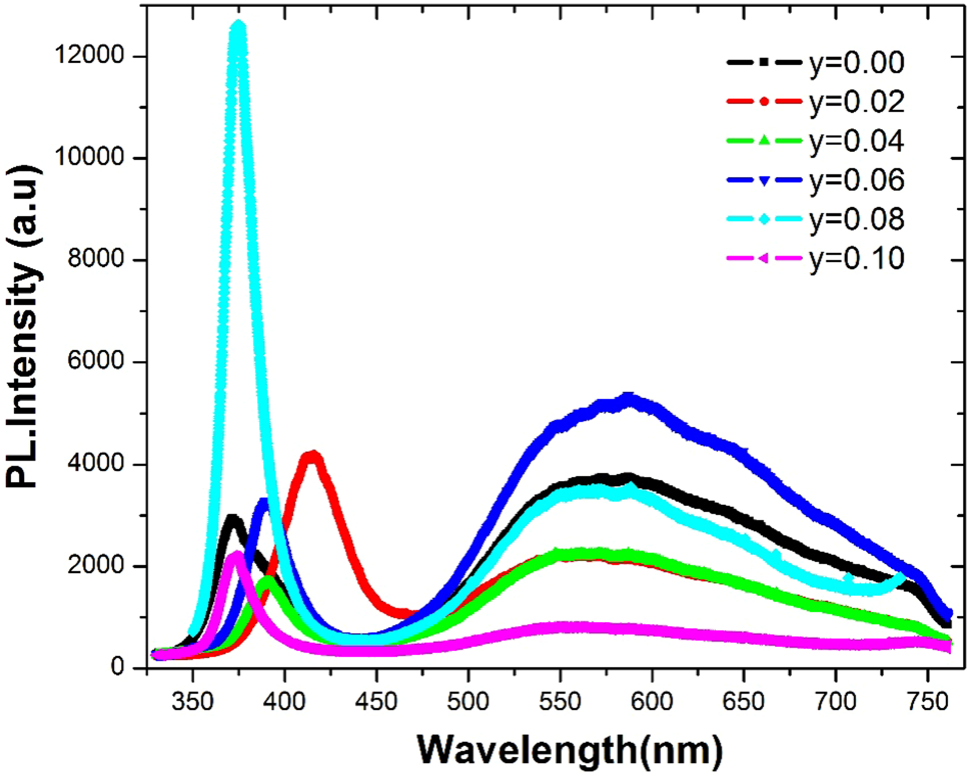
Room temperature PL spectra of Zn_1−*y*_C_*y*_O (0.00 ≤ *y* ≤ 0.10) nanorods.

**Figure 9:**
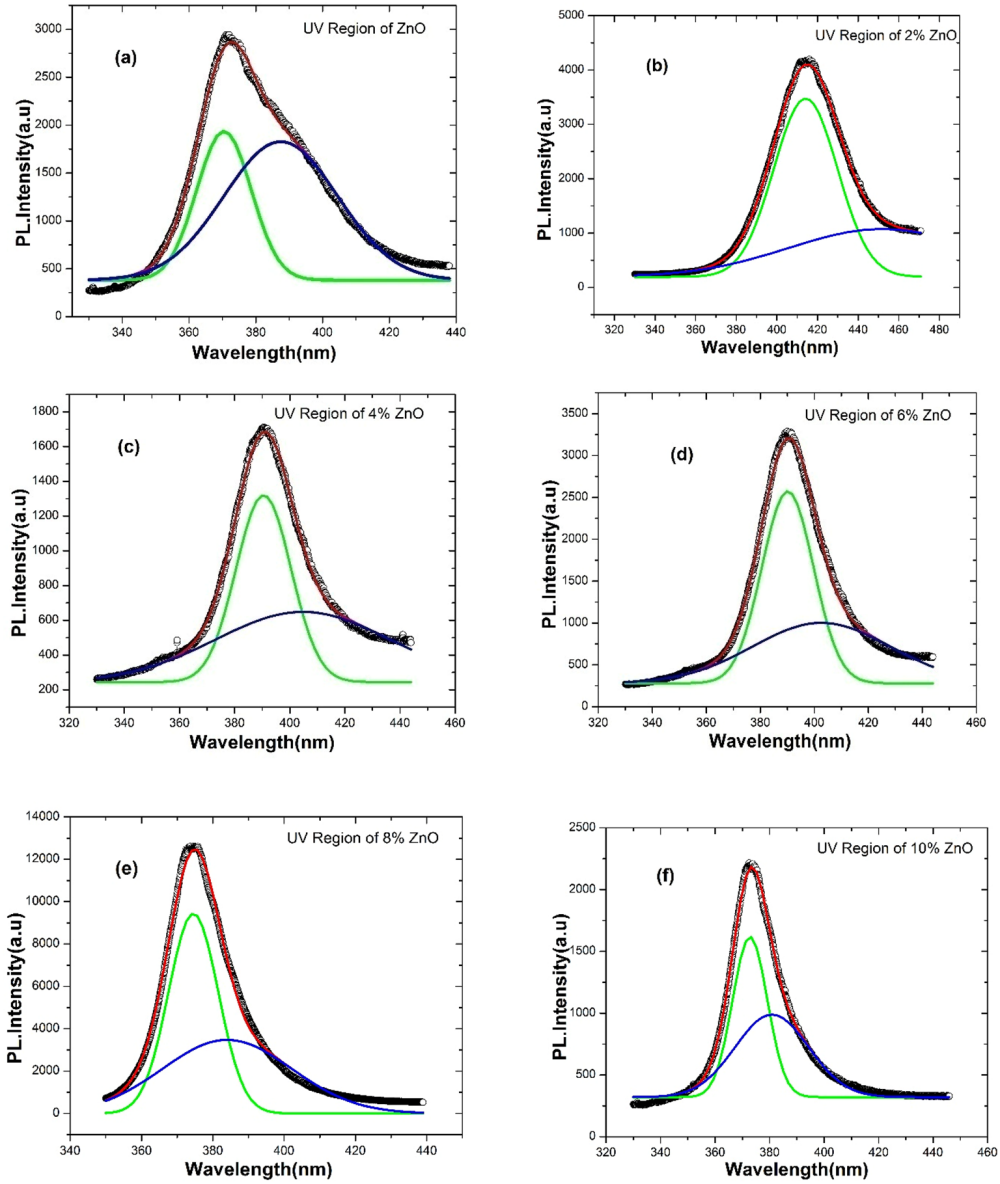
(**a**–**f**). Gaussian deconvolution of UV region of PL spectra for Zn_1−y_C_y_O (0.00 ≤ y ≤ 0.10) nanorods.

Figure 8 displays the room temperature photoluminescence (PL) spectra of Zn_1_-_y_C_y_O (0.00 ≤ y ≤ 0.10) nanorods in the range of 325–775 nm. Room temperature PL studies confirmed the presence of UV and visible broadband in all samples. The spectrum has two regions: ultraviolet (UV) and visible broadband (BB). PL spectra of ZnO and Zn_1_-_y_C_y_O (0.00 ≤ y ≤ 0.10) nanostructure samples have similar natures (shape) but exhibit different intensities and slight shifts in wavelengths. These variations in PL spectra due to changes in dopants can be attributed to the formation of various defects ^66^. The UV region, characteristic of ZnO, originates from free exciton (FX) emissions resulting from the combination of electron–hole (e–h) pair luminescence. The UV region typically describes the bandgap of the ZnO systems. The ratio of UV intensity (I_uv_) to the intensity of broadband (I_BB_) has been plotted in Fig. 5S(a). We observed a non-monotonic trend in the ratio of UV region and broadband intensities versus C concentrations. However, we found that the y = 0.08 sample has the highest value of the ratio, while the y = 0.06 sample has the lowest value. The intensity ratio of UV to BB in PL spectra of ZnO samples provides information about the total concentration of defects in each composition. Visible broadband reveals the development of local defects across the bandgap of ZnO. Usually, UV and visible BB regions have more than one emission and are repeatedly attributed to different defects in undoped and doped ZnO nanoscale systems ^67^. Figure 8 shows that PL intensity varies with increasing C-dopant, and UV emission for y = 0.08 composition has the largest peak intensity among C-doped samples. There is no significant change in the BB region with respect to wavelength, but there are changes in PL intensity. The PL intensity of 6% C doped ZnO is greater than that of pure ZnO. PL intensities of y = 0.02, 0.04, 0.08, and 0.10 doped ZnO samples are slightly less than the intensity of the pure ZnO sample. The intensity variations of UV and visible BB were observed in samples related to the concentration of possible defects in each composition at different concentrations. The variation in PL intensity observed in these C doped ZnO samples can be attributed to the presence of different types of defects (e.g. dopant related defects, oxygen and Zn vacancies, interstitial defects, surface defects) induced by the C dopant. The concentration of these defects plays a crucial role in modifying the radiative recombination pathways, resulting in the observed changes in PL intensity in both the UV and visible BB regions. Gaussian deconvolution of UV region PL spectra is shown in Fig. 9a–f for different compositions of C-doped ZnO samples. Gaussian fitting for the UV region has two types of transition energies attributed to free exciton transition (FX) and donor–acceptor pairs (DAP), respectively. The FX peak (i.e., green fitted peak in our case) is associated with near band edge (NBE) emission, corresponding to the free exciton recombination process ^68^. It suggests that NBE emission shifts toward lower energy due to carrier impurity and carrier phonon interactions, known as the band tailing effect ^69^. We plotted FX (in eV) versus C content, as shown in Fig. 5S(b). Overall, we found that the bandgap (i.e., FX peak position in the case of ZnO) of doped samples decreased compared to the undoped ZnO sample. However, the 2% C doped sample has the smallest bandgap among all samples, while the bandgap increased upon further doping of C content. Similar observations have been reported in other materials doped in ZnO structures ^70^. From the deconvoluted UV region of PL spectra, it can be argued that shallow acceptor states are generated above the valence band in 2% C-doped samples. This is the main reason for the decrease in bandgap compared to the undoped sample. With further increase in C content (e.g., for x = 0.04), we observed that the bandgap increases to y = 0.10. The increase in the bandgap for doped samples may be attributed to the enhancement of deep acceptor states within the valence band as the C content increases, leading to an overall increase in the bandgap. Similar effects have been reported by Liu et al. for Ga doping of ZnO thin films ^71^.

**Figure 10:**
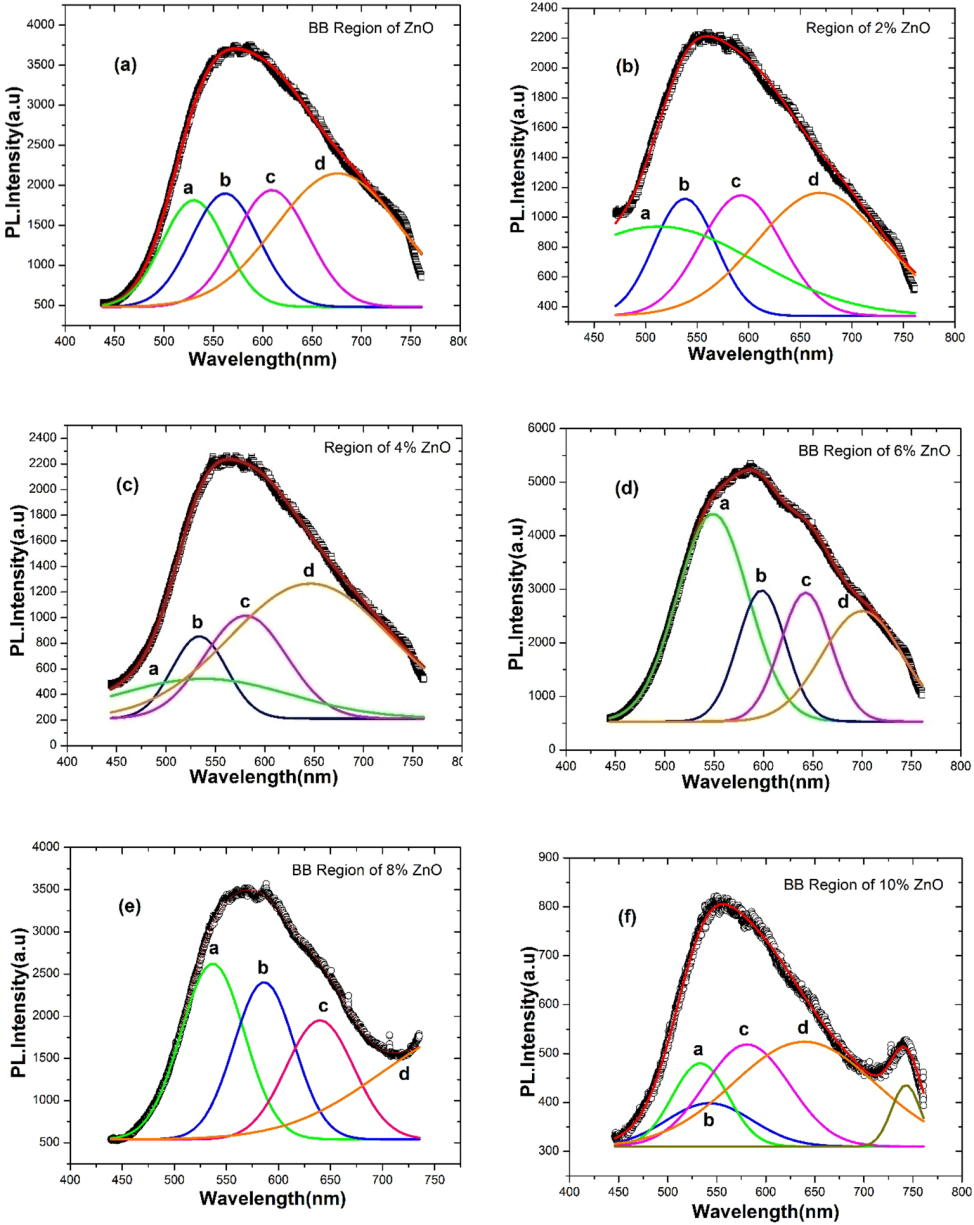
(**a**–**f**) Gaussian deconvolution of PL visible broad band spectra for Zn_1−y_C_y_O (0.00 ≤ y ≤ 0.10) nanorods on seeded Si substrate.

Our PL data revealed a visible BB with an asymmetric nature. This band must be symmetric by Gaussian deconvolution to identify possible defects within the bandgap. Figure 10a–f displays the visible BB emission (450–750 nm) of Zn_1_-_y_C_y_O (0.00 ≤ y ≤ 0.10) nanorods well resolved into four different symmetric Gaussian peaks. We calculated the peak positions of these fitted Gaussian peaks from the deconvoluted broadband PL data for the entire series of samples. The estimated centered peak position values are presented in Table 3S. These fitted peaks are attributed to transitions due to defect states. The fitted peak “**a**” may be ascribed to the electron transition from the donor levels due to zinc interstitials or oxygen vacancies to the valence band. The fitted peak “**b**” can be attributed to the transition from the conduction band to the V_Zn_ defect level. The fitted peak “**c**” may be associated with interstitial oxygen defects. The fitted peak “**d**” might resemble transitions from donor levels in the crystal (V_O_, Zn_i,_ and Ci) to C_Zn_ acceptor levels. Various types of defects are present here in our deconvoluted PL spectra according to the literature ^72^, but the nature of these defects has not been conclusively identified. Investigations into the origin of these defects are ongoing. Similarly, the deconvolution for broad visible band emission for Li–N and Li–F co-doped ZnO nanostructures have been reported previously ^54^. Green luminescence in ZnO is commonly observed ^73–75^, although the origin of this emission remains highly controversial. We discovered that deconvoluted peaks have binding energies related to various defects ^76^. The deconvolution of visible BB confirmed the existence of different defects at various energy levels within the bandgap of the ZnO system. More research is necessary, particularly at low temperatures, to fully grasp the broad visible band's real recombination mechanism because the microscopic interstitial and substitutional and antisite defects present in this C-doped ZnO nanorods system are difficult to interpret, and their locations within bandgap are still up for debate and controversy.

### Computational studies

**Figure 11:**
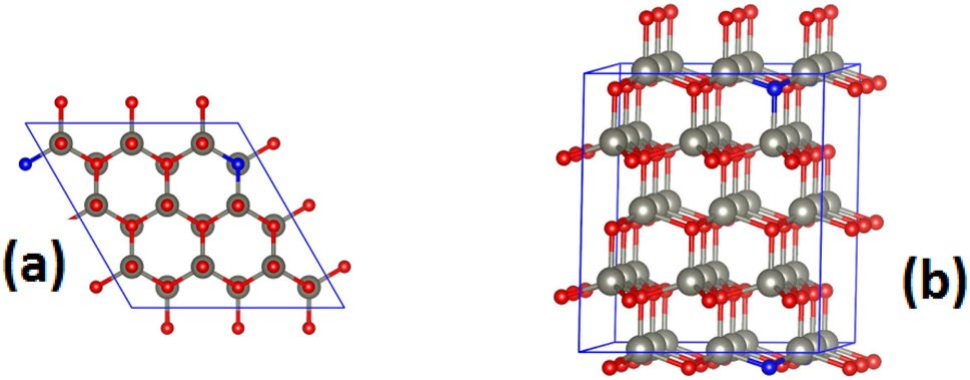
Top (**a**) and (**b**) side view of a 3 × 3 × 2 supercell of ZnO with one O replaced by C. The gray, red, and blue colored spheres represent Zn, O, and C atoms respectively.

We used Density Functional Theory (DFT) implemented in the Vienna Ab initio Simulation Package (VASP) to systematically investigate the structural, electronic, and magnetic properties of dilute C-doped ZnO nanorods. Cut-off energy of 600 eV is used for the plane-wave basis sets for both ZnO and C-doped ZnO nanorod systems, and the projector augmented-wave potentials are utilized as follows: Zr_PAW_PBE (3d10, 4S2), O_PAW_PBE (2S2, 2p4), and C_PAW_PBE (2S2, 2p2). A 3 × 3x3 Monkhorst–Pack k-mesh is employed for the 3 × 3x2 supercell of ZnO. All structures were optimized until the largest Hellmann–Feynman forces reached − 0.001 eV/Å. A unit cell of pristine ZnO is optimized in a hexagonal structure, resulting in lattice parameters a = b = 3.288 Å and c = 5.305 Å. Subsequently, a 3 × 3 × 2 supercell with a = b = 9.865 Å and c = 10.612 Å is modeled (as shown in Fig. 11) to obtain the dilute homogeneous C-doped ZnO samples. This supercell consists of 72 atoms (36 Zn and 36 O). As the diameter of the C-doped ZnO nanorods prepared in our experiment is several thousand Å, our calculations for single crystal ZnO are expected to accurately interpret the experiment.

To explain the magnetism observed in the dilute C-doped ZnO nanorod samples, all possibilities are considered in Table 1. The total energy (E), volume of the supercell (V), and magnetic moment (M) of pristine, O-vacancy, O-substituted with C, Zn-substituted with C, and C at the interstitial position in a 3 × 3x2 supercell of ZnO are computed and listed in Table 1. Replacement of O with C increases the energy of the system, indicating that the C–Zn bond is weaker than the Zn–O bonds. Although total energy calculations do not suggest O replacement with C, magnetism only occurs when C occupies the O or interstitial sites. The highest magnetic moment of 2 µB is observed for C at the O site. Our experiment shows a high magnetic moment for low C concentrations (i.e. y = 0.02 sample), so C occupies the O site rather than the interstitial site. We have examined two cases to consider changes in the magnetic moment with increasing C concentration. In the first case, the two nearest neighboring O atoms are replaced with C, while in the second case, the two farthest O atoms are replaced with C. Total energy calculations show that the first case is preferred by 0.691 eV over the second case. For the second case, the magnetic moment doubles, while for the first case, the magnetic moment does not change with increasing C concentration. Thus, in agreement with our experiment, our calculations indicate that the highest magnetic moment can be achieved with lower concentrations of C.

**Table 1:** Total energy (*E*), volume (V), and magnetic moment (*M*) for a 3 × 3 × 2 unit cell of pristine ZnO, O-substituted by C (C_O), Zn-substituted by C (C_Zn), C at the interstitial site (C_int_), and single O vacancy (O_vac_).

System	ZnO	C_O	C_Zn	C_*int*_	O_*vac*_
*E* (eV)	− 327.609	− 324.528	− 330.971	− 332.201	− 318.983
*V* (Å^3^)	894.33	896.82	887.50	912.20	884.86
*M* (*μ*_*B*_)	0.00	2.00	0.00	0.84	0.00

**Figure 12:**
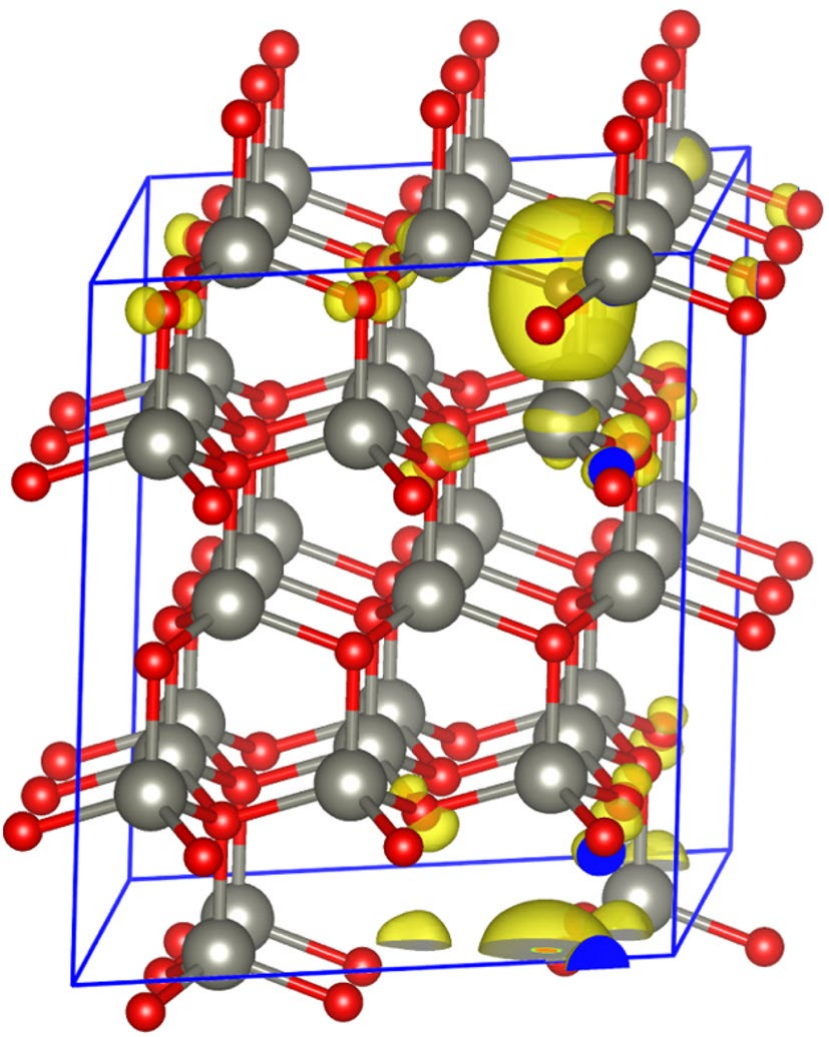
Spin charge density (ρ↓ − ρ↑ ) at the isosurface of 5 × 10^−3^ for the C-doped 3 × 3 × 2 supercell of ZnO.

**Figure 13:**
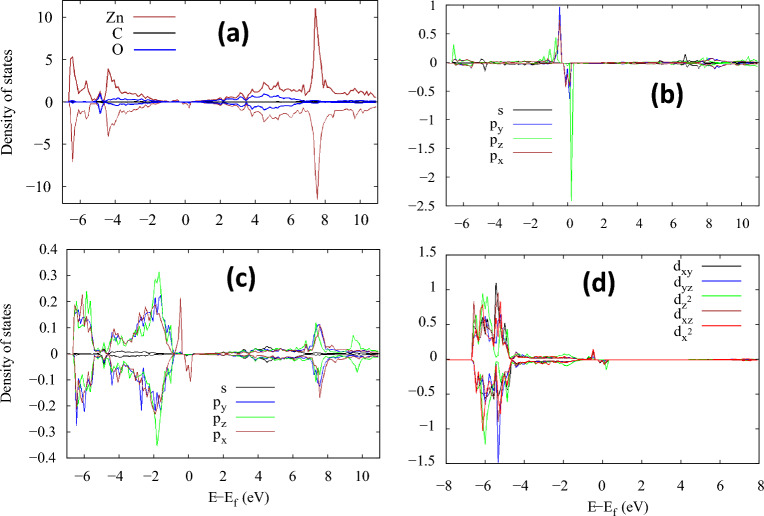
Spin-polarized projected density-of-state for (**a**) 3 × 3 × 2 supercell of C-doped ZnO showing contributions from Zn, C, and O, respectively. Contribution from C is negligible while that from Zn is dominant. (**b**) Spin-resolved density-of-states for C, (**c**) O nearest to C, and (**d**) Zn nearest to C.

To further discuss the origin of magnetism by O substitution with C in ZnO, we have displayed the spin-charge density of a single C-doped ZnO in Fig. 12. This configuration shows that the magnetic moment mainly arises from C and its neighboring O and Zn atoms. In Fig. 13, we present the projected density of states for C-doped ZnO in a 3 × 3x2 supercell. Figure 13a illustrates the contribution to the density of states from Zn, C, and O atoms. The Zn densities of states are dominant, while those of C are negligible due to their lower concentration. To clarify the contribution of C, O, and Zn to the magnetic moment in our system, partial density of states for C, O, and Zn are shown in Fig. 13b,c. This demonstrates that magnetism from C is dominant due to its p-orbitals. Similarly, the O p-orbitals also have some contribution, while in the Zn case; it comes from the d-orbitals only. Furthermore, we anticipate that if C substitutes O sites in the ZnO structure, the C-2p orbitals become localized and create two holes at each site, leading to enhanced *p–p* type interactions due to coupling and strong spin interactions between C atoms and carriers, which can sustain and stabilize the long-range order magnetic properties for spintronic applications.

### Magnetic measurements and discussion

**Figure 14:**
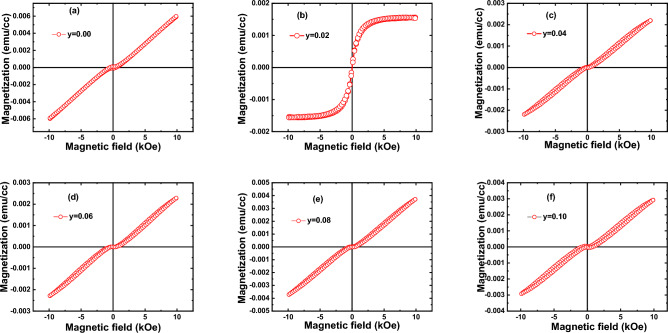
Room temperature M/H loop of Zn_1−*y*_C_*y*_O (0.00 ≤ y ≤ 0.10) on seeded Si substrate.

Room temperature DC magnetization measurements (i.e., magnetization (M) versus applied magnetic field (H), known as M/H curve) were performed for a bare Si-substrate and series of Zn_1−y_C_y_O (0.00 ≤ y ≤ 0.10) nanorod samples. The raw data (not shown here) were diamagnetic for all samples except the 2% C-doped ZnO sample, which displayed prominent ferromagnetic nature. After subtracting the background diamagnetic signal (due to the Si background effect) from the M/H loops, we observed weak ferromagnetic signals in the presence of paramagnetic signals except for y = 0.02 composition, as represented in Fig. 14a–f. Our magnetic loops confirmed the presence of coercivity and remanence in all samples. Thus, our samples are far from exhibiting superparamagnetic-type magnetization. The coercivity and remanence (remanent magnetization) were observed and are shown in the inset of the M/H curve of each sample. After subtracting the background effects, we observed a prominent strong s-shaped ferromagnetic signals curve in the y = 0.02 nanorods sample. From this observation and based on our DFT studies and XPS results, we can conclude that as more carbon is replaced by oxygen sites in the crystal structure of ZnO, reasonably strong ferromagnetic signals will be attained. We probably infer that the paramagnetic nature in our samples may be arising due to the combined or induvial effects of many other remaining defects calculated from PL and XPS studies.

Ferromagnetism exists in C-doped ZnO nanostructured systems but still has strong controversy in the literature from both the experimental and the theoretical points of view. Many groups claim that C substitutes O sites, while others argue that C occupies Zn sites in the hexagonal crystal structure. Based on the first possibility, in 2007, Pan et al*.* ^19^ reported that RTFM in the C-doped ZnO thin films prepared by PLD is due to the Zn-C configuration. Based on their calculated band structures, substituting C atoms at O sites generates holes in O2p states, coupled with the parent C2p localized spins through *p–p* hybridization. In 2008 Zhou et al. ^77^ observed RTFM in C-doped ZnO thin films associated with the chemical involvement of carbon via ion-implantation technique by substituting C on O sites. In 2010, Ye et al*.* ^78^ deduced that the generation of magnetic moment is a result of the displacement of an oxygen atom by a carbon atom. The magnetic moment declines when oxygen vacancy is present in the ZnO lattice. Furthermore, they found that with variation in carbon distribution, there is a significant change in magnetic moment due to the coexistence of antiferromagnetic and ferromagnetic interactions. Li et al. ^79^ experimentally observed that both a certain number of donor defects (Vo or/and Zni) and the net spin of Zn ions caused by the substitution of O by C are two key factors in inducing magnetic ordering in C-doped ZnO films were prepared by PLD. Nagare et al*.* ^80^ carried out spin-polarized electronic structure calculations ZnnOn-mCm (m = 1–2). They found that all systems with two carbon impurities show ferromagnetic interaction, except when carbon atoms share the same zinc atom as the nearest neighbor by hybridizing zinc 4 s with carbon 2p and oxygen 2p orbitals. This ferromagnetic interaction is predominantly mediated via π-bonds in the ring structures and through π- and σ-bonds in the three-dimensional structure.

In 2012, Subramanian et al. ^81^ demonstrated that RTFM in C-doped ZnO thin films is due to the charge transfer between Zn-4 s and C-2p orbitals. The long-range magnetic interaction is due to carbon–carbon interaction mediated by oxygen ions, i.e., hybridization between Zn and C species. Zheng et al. ^82^ theoretically studied the C-doped ZnO nanosheets and found that the C atoms tend to form a cluster around the Zn atom. The long-range FM coupling is mainly mediated by the O atoms. The mechanisms responsible for FM are the collective effects of a *p–p* coupling interaction. Using the first principal studies, Nayak et al*.* ^83^ established the two possible substitutional carbon impurities in ZnO (C_O_ and C_Zn_) and concluded that partially filled impurity bands in the minority spin channel mediate ferromagnetic interaction between the C_O_ impurities, i.e., two C_O_–C_O_ impurities in ZnO interact ferromagnetically, but the interaction is found to be short-ranged and anisotropic, much stronger within the hexagonal ab plane of wurtzite ZnO than along the c-axis. Mishra et al. ^84^ found experimentally and theoretically that the presence of Zn–C complexes surrounded by a ZnO matrix indicates that the ferromagnetic signature in carbon-doped ZnO nanoparticles arises from creating defects or developing oxy-carbon clusters. In 2018, Ngo et al*.* ^85^ experimentally demonstrated that migration of C atoms into ZnO crystal to substitute O vacancies C-Doped ZnO@C Core–Shell Nanostructures and proposed that the s–p and p–p hybridizations formed by C2p–Zn4s, and O2p–C2p orbitals are believed to cause ferromagnetism. Beltrán et al. ^47^ suggested that the presence of *C*_*O*_ defects, with carbon atoms on an oxygen site, is the likely source of the magnetic moments, which may interact ferromagnetically via the mediation of oxygen atoms. The formation of C–Zn–C bonds encourage the AFM interaction, and the formation of intrinsic defects induced by carbon atoms such as *V*_*O*_, *V*_*Zn,*_ and *C*_*Zn*_ do not have a crucial role in the FM signal. In contrast, few studies showed origin of ferromagnetism in C doped ZnO nano system may be due to incorporation of C at Zn sites i.e. C_Zn_ substitutional defects ^86–88^.

We found an experimentally strong ferromagnetic nature at a 2% C-doped ZnO nanorods system. Previous studies may support this observation where C-doping concentration, as low as 1%, ^77^ and other groups predicted 2% ^83^ and 3% ^47^ induces significant ferromagnetism in ZnO nanostructured systems. Our experimental and theoretical results agree with earlier reports, where researchers observe C substitutes at O sites in ZnO nanoscale system ^36,89^. We expect that if C substitutes O sites in the ZnO structure, the C-2p orbitals will be localized, and each site will create two holes, as theoretically predicted in our studies and earlier ^19^. The *p–p* interaction can lead to a strong spin interaction between C atoms and carriers, sustaining and stabilizing long ferromagnetic moments in these non-magnetic doped oxide-based nanoscale systems.

## Conclusion

We deposited seed layers of zinc acetate solutions on Si substrates via spin coating, creating Si-seeded substrates. Subsequently, we synthesized a series of Zn_1−y_C_y_O (0.00 ≤ y ≤ 0.10) samples using the dip coating technique. SEM analysis confirmed that a single layer of seeds is sufficient for the subsequent growth of nanostructures on the seeded Si substrates. XRD data revealed that the 2θ values of peaks shifted towards higher angles in the C-doped samples than the undoped ZnO nanorods sample, suggesting the substitution of O sites by C. The fitted peak obtained from the PL spectra may correspond to the bandgap of the ZnO system. The bandgap variation versus carbon concentration exhibited a non-monotonic trend, as evidenced by the photoluminescence (PL) data within the 3.00–3.35 eV range. The lowest bandgap was observed in the ZnO nanorods doped with 2% C. The presence of the 2TO vibrational mode, attributed to multiple phonons at 983 cm^−1^, was only detected in the C-doped samples, indicating the incorporation of C species into the ZnO structure. Interestingly, we observed a strong ferromagnetic nature in the y = 0.02 C-doped sample, while the other samples exhibited a ferromagnetic signal with a paramagnetic nature. Room temperature ferromagnetic hysteresis measurements confirmed the presence of coercivity and remanent magnetization in all doped samples. Based on DFT calculations, when C substitutes O sites in the ZnO structure, the C-2p orbitals become localized, creating two holes at each site. The O p-orbitals also contribute, while in the Zn case, it originates solely from the d-orbitals. We propose that the expected p-type conduction in C-doped ZnO nanorod samples is associated with C incorporation. The p–p interaction can lead to strong spin interaction between C atoms and carriers, thereby sustaining and stabilizing the long-range ordered magnetic moment in these initially non-magnetic systems.

### Supplementary Information

Supplementary Information.

## Data Availability

The datasets used and/or analyzed during the current study available from the corresponding author on reasonable request.
