# Supplementary material for: Defects mediated weak ferromagnetism in Zn1−yCyO (0.00 ≤ y ≤ 0.10) nanorods semiconductors for spintronics applications
Source: Sci Rep. 2023 Oct 10;13:17080. doi: 10.1038/s41598-023-44102-w (PMC10564763; doi:10.1038/s41598-023-44102-w)
Supplement: Supplementary file 1 — Supplementary Information. [file 41598_2023_44102_MOESM1_ESM.pdf]

Supplementary Information

Defects mediated weak ferromagnetism in $Zn_{1-y}C_yO$ $(0.00 \leq y \leq 0.10)$ nanorods semiconductors for spintronics applications

Saif Ullah Awan[a)1†] M. Tanveer Akhtar[2], Danish Hussain[3], Saqlain A. Shah[4], Syed Rizwan[5], Mohsin Rafique[6], Abdus Samad[7], M. Arshad[8]

1. Department of Electrical Engineering, College of Electrical & Mechanical Engineering (CEME), National University of Sciences and Technology (NUST), Islamabad 44000, Pakistan
2. Department of Physics, COMSATS Institute of Information Technology, Islamabad 44000, Pakistan.
3. Department of Mechatronics Engineering, NUST College of Electrical and Mechanical Engineering, National University of Sciences and Technology (NUST), Islamabad 44000, Pakistan
4. Department of Physics, Forman Christian College (University), Lahore, Pakistan
5. Physics Characterization and Simulations Lab, School of Natural Sciences (SNS), National University of Sciences and Technology (NUST), Islamabad 44000, Pakistan
6. Beijing Academy of Quantum Information Sciences, Beijing, China.
7. Department of Physics, University of Ulsan, Ulsan 44610, Republic of Korea.
8. National Centre for Physics, Shahdara Valley, Islamabad Pakistan

[†] saifullahawan@ceme.nust.edu.pk, ullahphy@gmail.com

1. Introduction:

Room temperature ferromagnetism (RTFM) in oxide based materials due to the action of certain structural and electronic defects such as cationic vacancies [1] and oxygen vacancies [2] is an important field of research. This is classified as *"defect ferromagnetism"* in the field of magnetism [3]. It is being recognized as an intriguing new area of magnetism where the ferromagnetism does not originate in parent materials but is generated by certain defects [4] that affect the electronic and magnetic properties at room temperature. The materials are expected to have applications in the field of spintronics if the role of defects can be understood and controlled finely. For this purpose ZnO is a promising material due to its multiferroic behavior. In this study one-dimensional (1-D) nanostructures [5, 6] of ZnO will be prepared and doped with non-magnetic dopants such as carbon and characterized for their structural, optical, electronic and magnetic properties.

During the last decades nanomaterials have received a lot of attention due to their high surface to volume ratio, quantum confinement effects and high surface energies which have opened up possibilities of exciting breakthroughs in science and technology [7]. Size and dimension play a vital role in determining material properties at the nanoscale. The study of low-dimensional systems (i.e. with characteristic dimensions of 1–100 nm) has become one of the most important and exciting fields of physics [7, 8]. Quantum confinement [8] in low dimensional semiconductors has led to exciting new properties, resulting from dramatic changes in the density of states. If the motion of electrons is confined predominantly in one dimension, the nanostructure is considered to be one dimensional (1D) for example nanobelts, nanorods and nanowires [9, 10]

Zinc oxide (ZnO) belongs to the II-VI group in the periodic table is a wide and direct band gap semiconductor energy of ~3.34 eV at room temperature [11] with a high efficiency of UV emission. ZnO is a compound semiconductor whose ionicity resides at the borderline between covalent and ionic semiconductor

[12]. ZnO structures found in two structures, one is hexagonal wurtzite[13, 14] and other is cubic Zincblende[15]. Wurtzite structure found common and most stable form due to surrounding conditions although Zincblende structure is only stable when a cubic structure on a substrate. In both cases, the zinc and oxide focuses are tetrahedral. Hexagonal and Zincblende polymorphs have no reversal symmetry. At ambient conditions, the thermodynamically stable phase is wurtzite. The wurtzite structure has a hexagonal unit cell with two lattice parameters, *a* and *c*, in the ratio of c/a=1.633. [12]. This material finds a host of applications in widely used in production of blue, green and white LEDs and laser diodes. The advantage it has over comparable materials e.g. GaN (another wide band gap semiconductor) is its large exciton binding energy (~60 meV) more than twice the room temperature energy of 25meV[16, 17]. The transparency of ZnO in the visible range is maintained even at very high carrier concentrations, making ZnO a promising material to be used as a transparent conductor. ZnO is one of the most important materials used in material research for a variety of applications such as electroluminescent devices, solar cells, optoelectronics, transparent electronics, and spintronics devices. ZnO has a tendency to grow in interesting morphology not only due to different surface energies but also due to surface polarity or chemical activity. Most of the group-II-VI binary compound semiconductors crystallize in either cubic zinc-blend or hexagonal wurtzite structure where each anion is surrounded by four cations at the corners of a tetrahedron, and vice versa. The tetrahedral coordination in ZnO results in a non-central symmetric structure and consequently piezoelectricity and pyroelectricity [12]. ZnO has the type of bonding which is to great extent (Zn^{+2}--O^{-2}) ionic with the relating radii of 0.074nm for Zn^{2+} and 0.140nm for O^{-2} ionic states[18].

1. S. U. Awan, S.K.H., M. F. Bertino, and G. H. Jaffari, J. Appl. Phys., 2012. **112**: p. 103924.
2. Lubna R Shah, B.A., Hao Zhu, WGWang,Y Q Song, H W Zhang, S I Shah and J Q Xiao, J. Phys.: Condens. Matter 2009. **21**: p. 486004.
3. Ogale, S.B., Adv. Mater. , 2010. **22**: p. 3125.
4. N. H. Hong, J.S., N. T. Huong, N. Poirot, and A. Ruyter Phys. Rev. B, 2005. **72**: p. 045336.
5. Seung Chul Lyu, Y.Z., and Cheol Jin Lee, Chem. Mater. , 2003. **15**: p. 3294.
6. Vugt, D.V.a.L.K.v., Nanoscale, 2011. **3**: p. 2783.
7. Moriarty, P., Rep. Prog. Phys. , 2001. **64**: p. 297.
8. Dana A. Schwartz, N.S.N., Quyen P. Nguyen, Jason M. Parker, and Daniel R. Gamelin, J. AM. CHEM. SOC. , 2003. **125**: p. 13205.
9. Shaw, J.W.L.a.J.M., J. Phys. D: Appl. Phys. , 2011. **44**: p. 303001.
10. Ross, F.M., Rep. Prog. Phys. , 2010. **73**: p. 114501.
11. Y. Chen, D.M.B., H.-J. Koh, K.-T. Park, K. Hiraga, Z.-Q. Zhu, and T. Yao,, J. Appl. Phys., 1998. **84** p. 3912
12. Ü. Özgür, Y.I.A., C. Liu, A. Teke, M. A. Reshchikov, S. Doğan, V. Avrutin, S.J. Cho, and H. Morkoç, J.Appl.Phys., 2005. **98**: p. 041301.
13. Guo, L., et al., *Regularly shaped, single-crystalline ZnO nanorods with wurtzite structure.* Journal of the American Chemical Society, 2002. **124**(50): p. 14864-14865.
14. Lee, H.-J., et al., *Study of diluted magnetic semiconductor: Co-doped ZnO.* Applied Physics Letters, 2002. **81**(21): p. 4020-4022.
15. Ashrafi, A.A., et al., *Growth and characterization of hypothetical zinc-blende ZnO films on GaAs (001) substrates with ZnS buffer layers.* Applied Physics Letters, 2000. **76**(5): p. 550-552.
16. T. Fukumura, Z.J., M. Kawasaki, T.Shono, T. Hasegawa, S. Koshihara, H. Koinuma, Appl. Phys. Lett., 2001. **78**: p. 958.
17. A. Tiwari, C.J., A.Kvit, D. Kumar, J.F. Muth, J. Narayan,, Solid State Commun. , 2002. **121**: p. 371.
18. Kang, H.S., et al., *Structural, electrical, and optical properties of p-type ZnO thin films with Ag dopant.* Applied Physics Letters, 2006. **88**(20): p. 202108-202108-3.

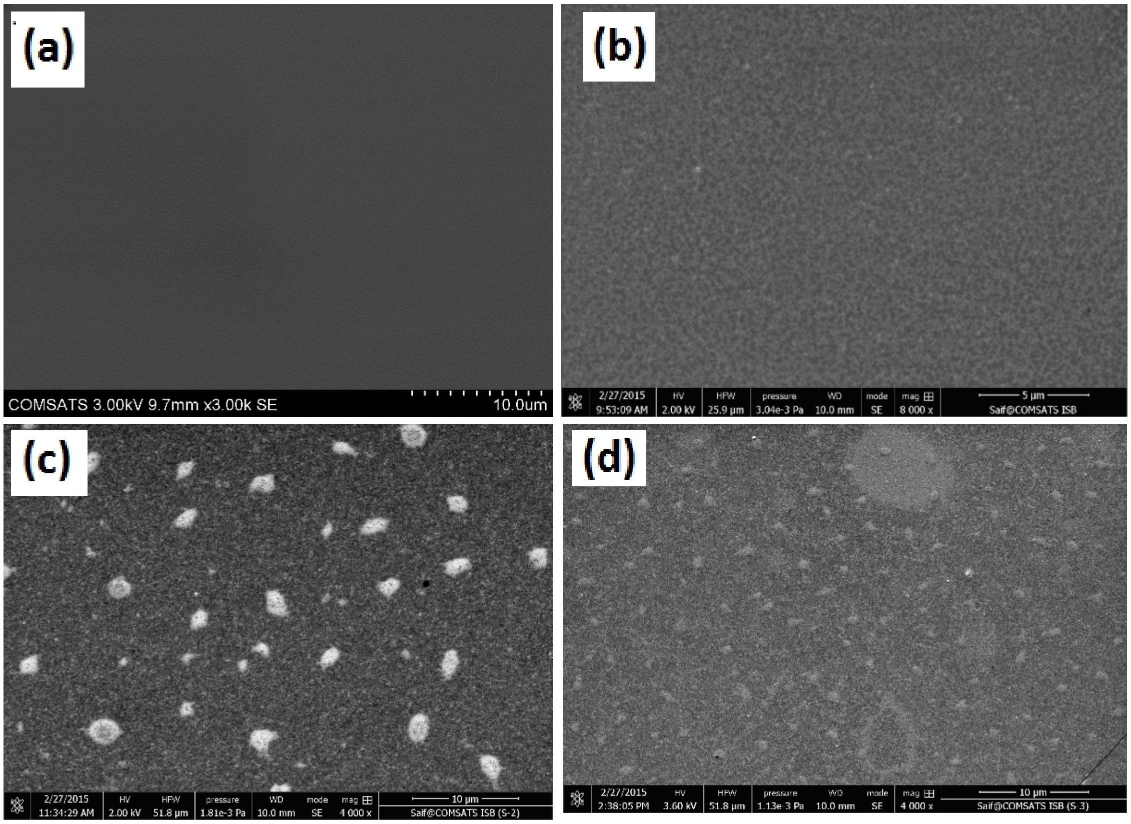

Fig.1S: SEM image of (a) pure silicon substrate, (b) 1-layer seeded Si, (c) 3-layers seeded Si, and (d) 7-layers seeded Si.

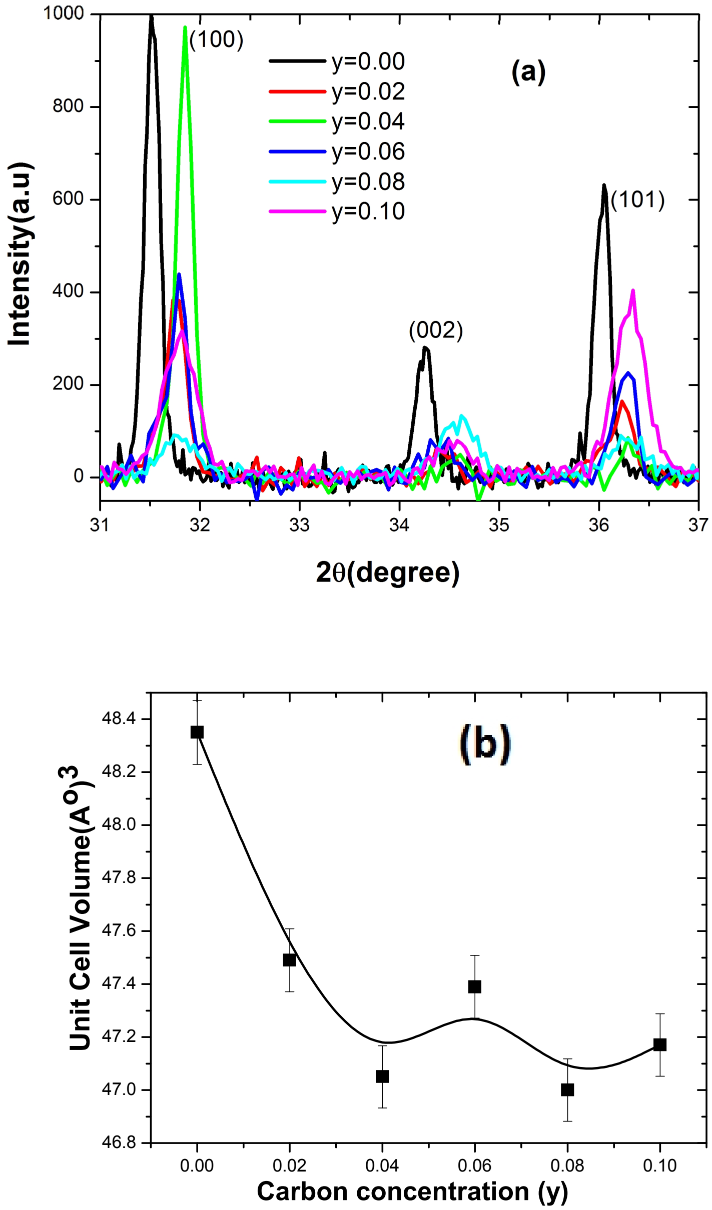

Fig.2S: XRD pattern of $Zn_{1-y}C_yO$ ($0.00 \leq y \leq 0.10$) nanorods **(a)** 2θ shifting of (100), (002), and (101) planes at ordinary intensity for entire series of samples, **(b)** variation of unit cell volume against C content.

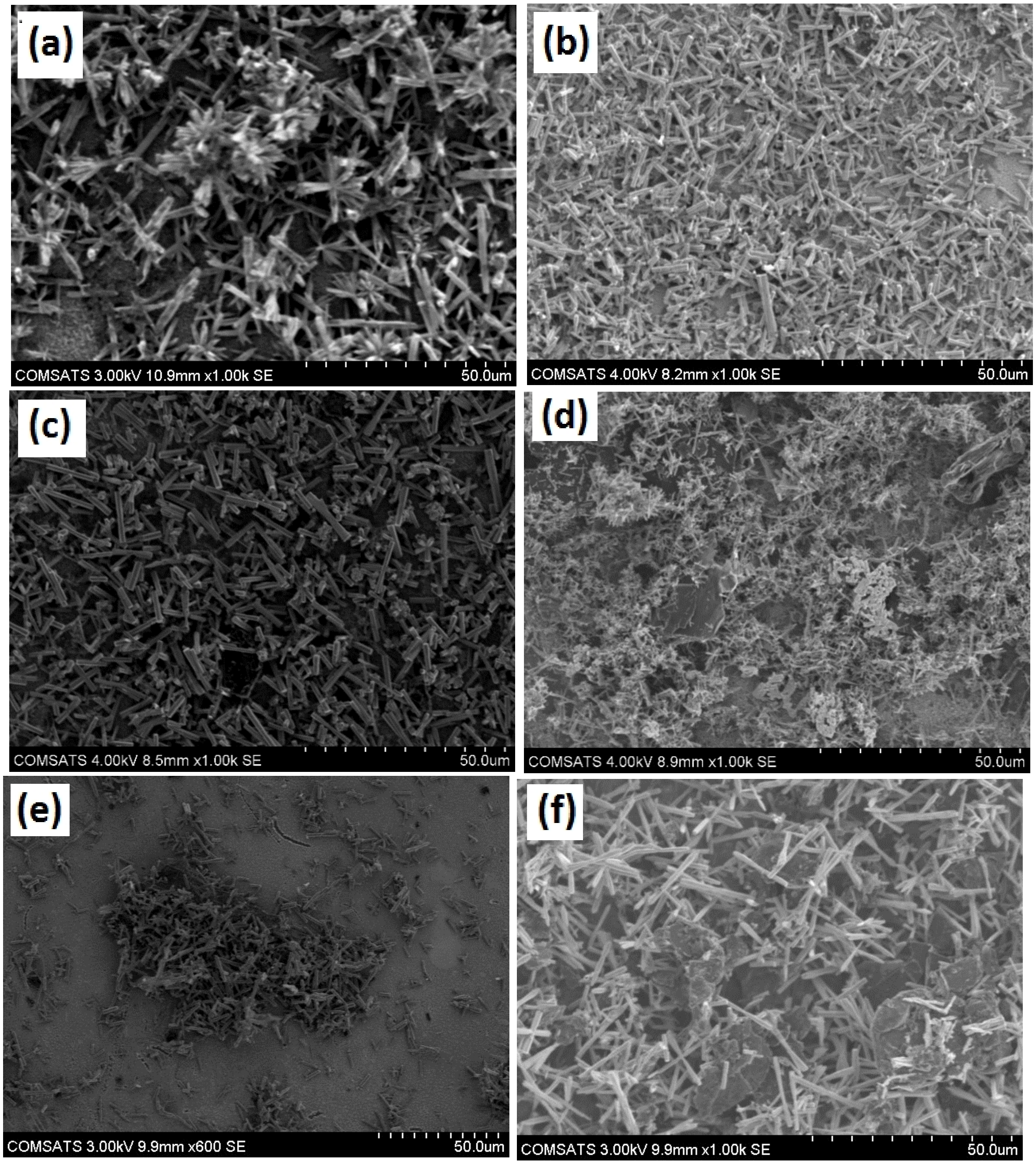

Fig.3S: Low resolution (i.e. at 50μm scale) SEM images of (a) pure, (b) 2%, (c) 4%, (d) 6%, (e) 8%, and (f) 10% C doped ZnO nanorods samples on seeded Si substrate.

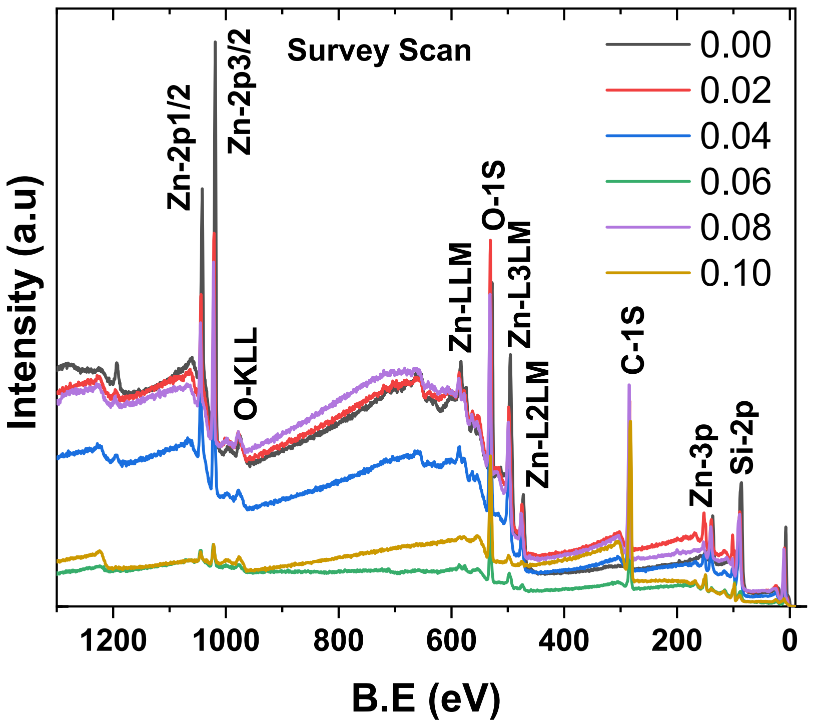

Fig.4S: Low resolution XPS Survey spectra of a series $Zn_{1-y}C_yO$ ($0.00 \leq y \leq 0.10$) nanorods on seeded Si substrate.

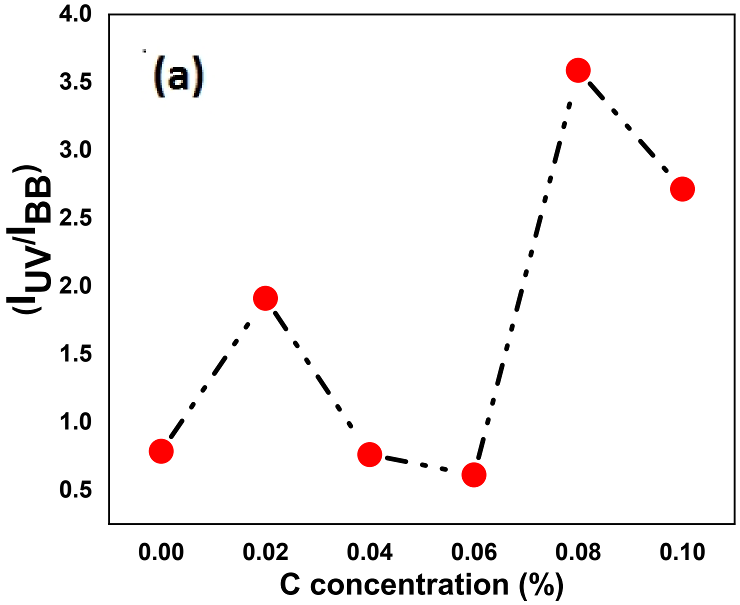

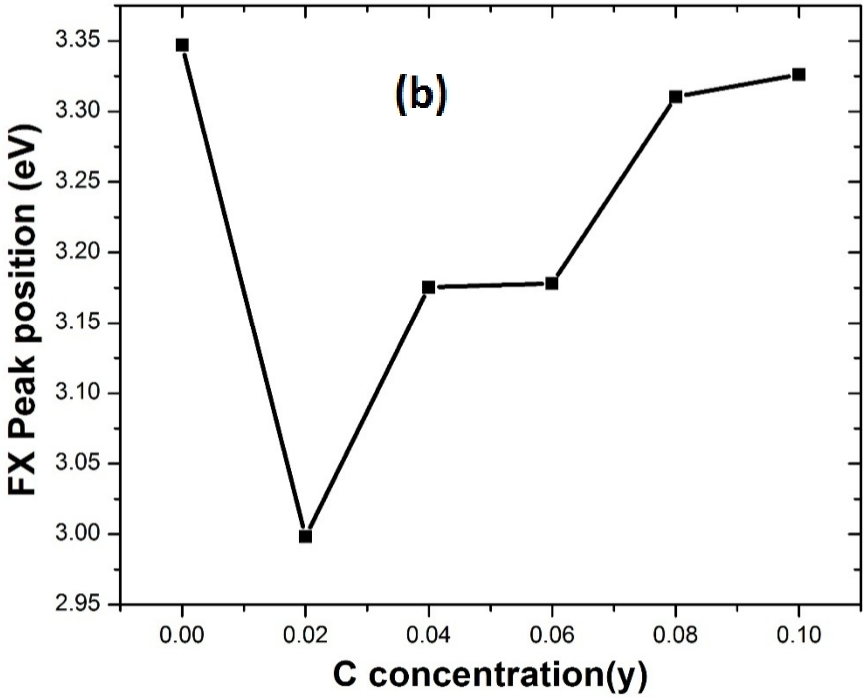

Fig. 5S. (a) Ratio of variation in PL intensity versus C percentage **(b)** FX peaks position as a function of C concentration for the series of $Zn_{1-y}C_yO$ ($0.00 \le y \le 0.10$) nanorods.

Table-1S: *d*-values, Lattice parameters and crystallite sizes of series of $Zn_{1-y}C_yO$ ($0.00 \leq y \leq 0.10$)

**Samples**	**2θ (degree)**	**2θ (degree)**	**2θ(degree)**	**$d_{(100)}$ (A^o)**	**$d_{(002)}$ (A^o)**	**$d_{(101)}$ (A^o)**	**Lattice Parameters (A^o)**	**Crystallite Size (nm)**	**Unit Cell Volume $(A^o)^3$**
	**(100)**	**(002)**	**(101)**						
**y=0.00**	31.51	34.26	36.05	2.830	2.614	2.488	a=3.268<br>b=3.268<br>c=5.228	38	48.35
**y=0.02**	31.73	34.55	36.23	2.816	2.593	2.476	a=3.252<br>b=3.252<br>c=5.186	46	47.49
**y=0.04**	31.85	34.61	36.29	2.806	2.588	2.473	a=3.240<br>b=3.240<br>c=5.176	34	47.05
**y=0.06**	31.79	34.49	36.29	2.811	2.597	2.473	a=3.246<br>b=3.246<br>c=5.194	46	47.39
**y=0.08**	31.86	34.62	36.42	2.805	2.588	2.464	a=3.238<br>b=3.238<br>c=5.176	26	47.00
**y=0.10**	31.82	34.58	36.34	2.808	2.591	2.469	a=3.242<br>b=3.242<br>c=5.182	30	47.17

Table-2S: The wave numbers (cm^{-1}) of first and second ordered Raman spectra were measured for entire series of $Zn_{1-y}C_yO$ ($0.00 \leq y \leq 0.10$) nanorods samples.

Modes	$y = 0.00$	$y = 0.02$	$y = 0.04$	$y = 0.06$	$y = 0.08$	$y = 0.10$
E_2^{High}	436	436	436	436	436	436
**Si**	520	520	520	520	520	520
**A_1(LO)**	579	579	579	-	-	-
**TA+TO**	-	-	-	620	620	620
**TA+LO**	671	671	671	682	682	682
**TA+LO**	851	851	851	837	837	837
**2TO (multiphonons)**	-	983	983	983	983	983
**2LO**	1140	1140	1140	1137	1137	1137

Table-3S: Peak positions of deconvoluted data of the broad band PL spectra of $Zn_{1-y}C_yO$ ($0.00 \le y \le 0.10$) samples on seeded Si substrate.

**Peaks (nm)**	**y=0.02**	**y=0.02**	**y=0.04**	**y=0.06**	**y=0.08**	**y=0.10**
a	531	505	524	546	539	531
b	563	539	535	599	584	579
c	607	593	578	642	639	638
d	673	667	642	701	730	744
